# Allelic Expression of Deleterious Protein-Coding Variants across Human Tissues

**DOI:** 10.1371/journal.pgen.1004304

**Published:** 2014-05-01

**Authors:** Kimberly R. Kukurba, Rui Zhang, Xin Li, Kevin S. Smith, David A. Knowles, Meng How Tan, Robert Piskol, Monkol Lek, Michael Snyder, Daniel G. MacArthur, Jin Billy Li, Stephen B. Montgomery

**Affiliations:** 1 Department of Pathology, Stanford University School of Medicine, Stanford, California, United States of America; 2 Department of Genetics, Stanford University School of Medicine, Stanford, California, United States of America; 3 Department of Computer Science, Stanford University School of Medicine, Stanford, California, United States of America; 4 Analytic and Translational Genetics Unit, Massachusetts General Hospital, Boston, Massachusetts, United States of America; 5 Program in Medical and Population Genetics, Broad Institute of Harvard and MIT, Cambridge, Massachusetts, United States of America; Georgia Institute of Technology, United States of America

## Abstract

Personal exome and genome sequencing provides access to loss-of-function and rare deleterious alleles whose interpretation is expected to provide insight into individual disease burden. However, for each allele, accurate interpretation of its effect will depend on both its penetrance and the trait's expressivity. In this regard, an important factor that can modify the effect of a pathogenic coding allele is its level of expression; a factor which itself characteristically changes across tissues. To better inform the degree to which pathogenic alleles can be modified by expression level across multiple tissues, we have conducted exome, RNA and deep, targeted allele-specific expression (ASE) sequencing in ten tissues obtained from a single individual. By combining such data, we report the impact of rare and common loss-of-function variants on allelic expression exposing stronger allelic bias for rare stop-gain variants and informing the extent to which rare deleterious coding alleles are consistently expressed across tissues. This study demonstrates the potential importance of transcriptome data to the interpretation of pathogenic protein-coding variants.

## Author Summary

Gene expression is a fundamental cellular process that contributes to phenotypic diversity. Gene expression can vary between alleles of an individual through differences in genomic imprinting or *cis*-acting regulatory variation. Distinguishing allelic activity is important for informing the abundance of altered mRNA and protein products. Advances in sequencing technologies allow us to quantify patterns of allele-specific expression (ASE) in different individuals and cell-types. Previous studies have identified patterns of ASE across human populations for single cell-types; however the degree of tissue-specificity of ASE has not been deeply characterized. In this study, we compare patterns of ASE across multiple tissues from a single individual using whole transcriptome sequencing (RNA-Seq) and a targeted, high-resolution assay (mmPCR-Seq). We detect patterns of ASE for rare deleterious and loss-of-function protein-coding variants, informing the frequency at which allelic expression could modify the functional impact of personal deleterious protein-coding across tissues. We demonstrate that these interactions occur for one third of such variants however large direction flips in allelic expression are infrequent.

## Introduction

**Figure 1:**
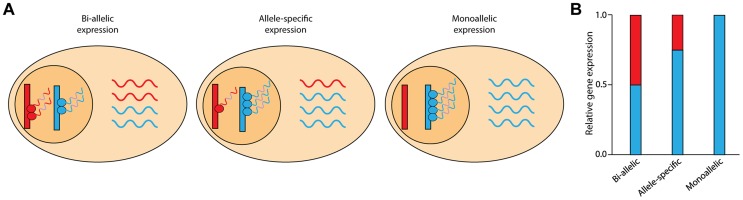
Schematic of allele-specific expression. (**A**) The two chromosomal copies (alleles) of a gene are shown in red and blue. In most cases, both alleles are transcribed; this is known as bi-allelic expression (left panel). In the case of allele-specific expression (middle panel), one allele exhibits greater expression than the other allele. When only one allele of a gene is actively transcribed, gene expression is termed monoallelic expression (right panel). (**B**) RNA-Seq reads across heterozygous sites can discriminate between the two alleles and quantify the relative abundance of expression. Although the relative gene expression levels may be similar, the allelic ratios can vary.

Recent genome sequencing studies have highlighted that healthy individuals carry multiple loss-of-function and rare deleterious variants whose interpretation is expected to inform individual disease risk and facilitate precision medicine [1]–[3]. However, accurate interpretation of these variants remains a considerable challenge as phenotypic effects remain difficult to predict. Furthermore, even when a specific function can be ascribed to a genetic variant, the variable penetrance and trait expressivity of genetic variants may yield important differences. In this respect, an important modifier of a coding allele's effect is its level of expression (Figure 1). This type of modification is likely to have considerable impact on interpretation of coding variant effects as genetic analyses of gene expression have reported that allele specific expression (ASE) influences at least 30% of genes for any given cell type [4], [5] and variability in allelic expression of pathogenic coding alleles has already been implicated in contributing to clinical variability for several diseases [6]–[10]. However, the degree to which deleterious and loss-of-function coding variants, routinely found through individual exome and genome sequencing, are allelically-expressed across multiple tissue types remains unexplored.

In this study we investigated patterns of gene expression and ASE for rare deleterious and loss-of-function variants across multiple tissues using both RNA-Seq and mmPCR-Seq, a targeted and high-resolution sequencing assay for measuring allelic ratios [11]. A major advantage of mmPCR-Seq is that it uncouples a gene's expression level, which can characteristically vary across tissues, from the power to measure allele-specific expression. Using this approach, we obtain 1000s of reads per heterozygous site per tissue to robustly quantify ASE. By comparing patterns of gene expression to allelic expression, we observed higher variability of allelic expression between tissues suggesting that expression level alone may be insufficient to predict the exposure of a damaging allele. Furthermore, we report patterns of ASE across tissues for both rare deleterious and loss-of-function protein-coding variants. These results demonstrate the extent to which regulatory variation can modify the functional impact of protein-coding variation across tissues, as well as the importance of using ASE for the interpretation of heterozygous variants in clinical sequencing analyses.

## Results and Discussion

### Collection of Deleterious and Loss-of-Function Variants

To map patterns of ASE for deleterious and loss-of-function coding variants, we sequenced the exome from two tissues (frontal lobe and small intestine) and RNA from ten tissues (cerebellum, frontal lobe, pancreas, stomach, small intestine, colon, heart, lungs, liver, and skeletal muscle) from a single individual. From the exome data, we identified 51,875 SNPs, of which 45,058 had consistent genotypes across tissues and were defined as “high-confidence” variants (Table S1). We identified 2,767 high-confidence variants that are private and not previously found in dbSNP [12], the 1000 Genomes Project [13], or the NHLBI Exome Sequencing Project (ESP) [14] (Table S2). Of these, 91 were heterozygous derived nonsynonymous variants classified by Sift [15] and Polyphen [16] as “damaging” and “deleterious”, respectively. Complementing these variants, we identified 106 SNPs that introduce premature stop-codons in exons, of which 75 SNPs were predicted to cause complete loss of function of all known transcripts using previously described prediction methods [1].

### Quantification of Allele-Specific Expression by RNA-Seq and mmPCR-Seq

We performed RNA sequencing (RNA-Seq) for each tissue (Figures S1, S2, S3) and intersected this data with high-confidence heterozygous variants to identify ASE patterns (Figure S4). ASE was determined on a per-heterozygote per-tissue basis using a binomial test where *p* is the empirical probability that a reference allele maps to the genome compared to a non-reference allele across all sites (Figure S5). Quality control filtering (by depth, p-value, bi-allelic expression and intragenic location) was performed to identify high-confident ASE sites across all tissues (Figure S6 and Table S3). The detailed method is available at http://montgomerylab.stanford.edu/resources.html.

The measurements of ASE by RNA-Seq are influenced by the depth of coverage of a gene in the assayed tissue [17], introducing challenges for ASE comparisons across tissues where genes are characteristically differentially expressed. To more accurately quantify ASE, we also applied our recently developed method that couples microfluidics-based multiplex PCR and next generation sequencing (mmPCR-Seq) [11]. We applied this technique to 74 deleterious, 50 nonsense and 205 control variants (Figure S7). Seventeen deleterious and 25 nonsense sites were excluded because they showed no evidence of expression in any of the ten tissues. For each tissue, we performed two technical replicates and mapped the merged sequence reads since we target-sequenced specific loci (Figure S8, S9). We applied the same pipeline and filters to detect ASE as those used for RNA-Seq. We further evaluated the correlation of effect size between technical replicates and observed high technical reproducibility (Figure S10). The small intestine and skeletal muscle have the greatest reproducibility (Pearson Correlation, *R*>0.93). The tissue with the lowest reproducibility is the pancreas (*R*>0.70), which contains a high concentration of nucleases and other enzymes that can degrade RNA. The variability of effect size between the replicates was also quantified for each tissue at varying read depths (Figure S11). As expected, sites with higher read depths have less variability between replicates. With the exception of the pancreas and frontal lobe, which are two tissues known to have low RNA quality post-mortem. Regardless, the variability of allelic ratios between replicates was well below 0.2 across all samples read depths. For tested sites, mmPCR-Seq provided greater depth and power to detect ASE and in many cases facilitated estimates for sites immeasurable without extreme RNA-seq coverage (Figures S12, S13). For instance, for 598 measurements which had no reads with RNA-Seq, we obtained an average of 2639 reads for mmPCR-Seq. Furthermore, only 73 measurements had greater than 100 reads for RNA-Seq compared to 817 for mmPCR-Seq.

### Differential Gene and Allele-Specific Expression

**Figure 2:**
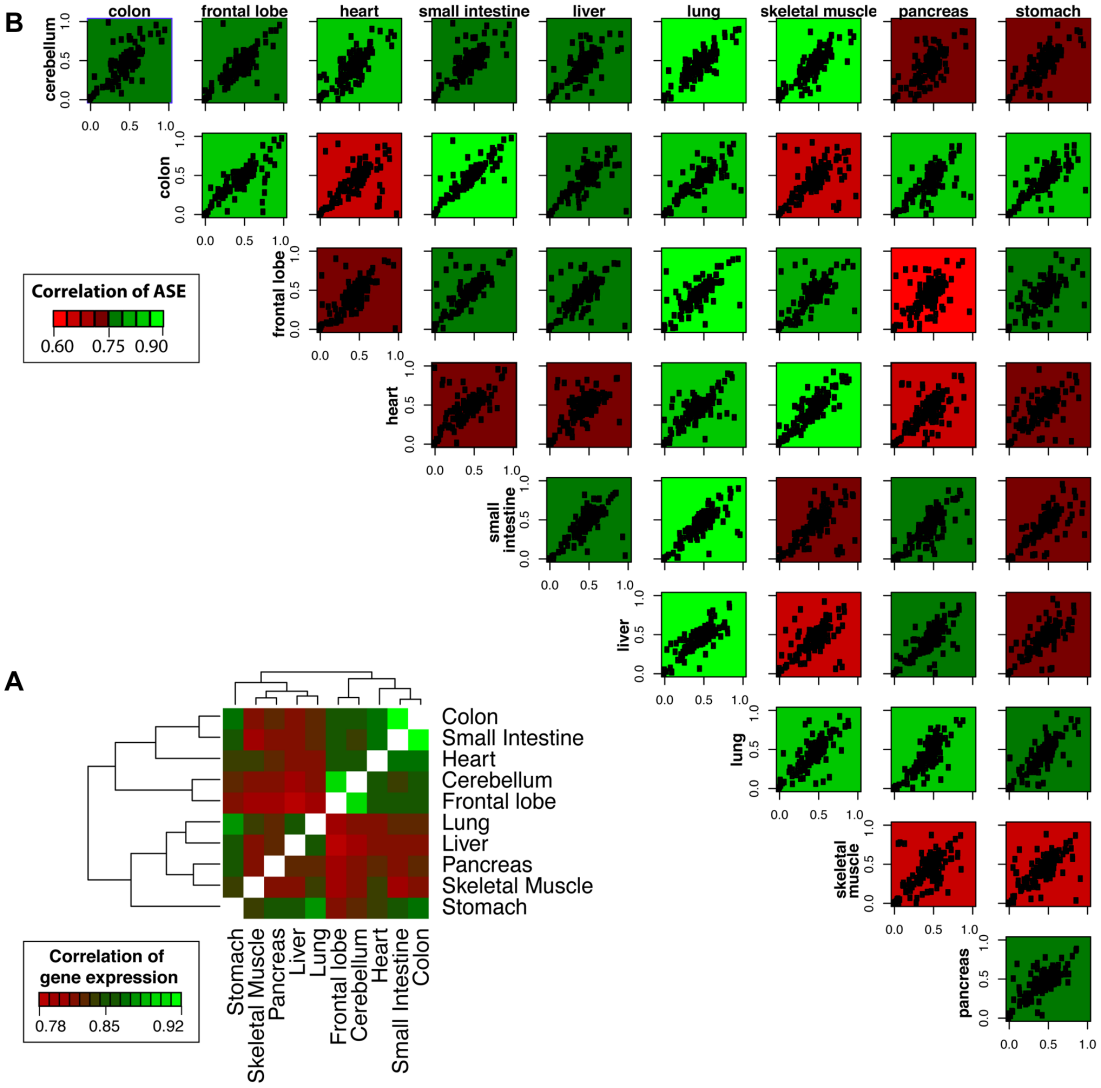
Correlation of gene expression and allelic ratios across ten somatic tissues. (**A**) Shared patterns of gene expression were detected for tissues with shared functional roles or embryonic origins. For example, the small intestine and colon are both digestive system organs derived from the endoderm and have a high degree of pairwise correlation (Spearman Correlation, *R* = 0.92). Likewise, the frontal lobe and cerebellum, which are both vital tissues nervous system derived from the ectoderm, have a high degree of shared expression (*R* = 0.91). The hierarchical clustering was generated using pairwise Spearman correlation coefficients of FPKM expression values for all genes. (**B**) Shared patterns of ASE were detected by mmPCR-Seq. The concordance of ASE between tissues does not as strongly reflect the relationships seen for shared gene expression or shared embryonic origin. The allelic ratio is calculated as the alternate allele reads divided by the total reads. Each data point represents a single heterozygous site tested for ASE with a total read depth greater than 200. The plots are colored by the degree of correlation of allelic bias between the pairwise tissues. These results indicate that relationships of allelic expression across tissues are much more complex than those of total expression level.

We next examined the sharing of gene expression and allelic effects across different tissues. Shared patterns of gene expression are detectable for tissues with shared functional roles or embryonic origins (Figure 2A, inset). For instance, the small intestine and colon, which are both digestive system organs derived from the endoderm layers, have a high degree of correlation (Spearman Correlation, *R* = 0.92). Likewise, the frontal lobe and cerebellum, which are both neural tissues derived from the ectoderm, have a high degree of shared gene expression (*R* = 0.91). To test the degree of correlation of allelic expression across tissues, we measured concordance of allelic ratios between pairwise tissues using the high-depth mmPCR-Seq data. Here, allelic ratios are defined as the ratio of the non-reference allele to the sum of the non-reference allele and the reference allele. We observed that the concordance of ASE between tissues does not as strongly reflect the relationships seen for shared gene expression or shared embryonic origin (Figure 2B). The range of pairwise tissue correlation for allelic effects ranges between 0.46 and 0.80, with the small intestine and colon having the most similarity (*R* = 0.80). We also compared in detail the pairwise correlation coefficients for expression and allelic ratios for tissue pairs of highly similar embryonic origin (Figure S14). We compared two neural tissues (frontal lobe and cerebellum) both derived the ectoderm and two intestinal tissues (small intestine and colon) both derived from the mesoderm. Irrespective of read depth and sequencing technology, the correlation of expression for tissues is consistently greater than the correlation of allelic effects across tissues. This observation suggests that allelic effects exhibit more variability than gene expression across tissues.

We also investigated the sharing of monoallelic expression across tissues (Figure 1). We identified five genes (*NDN*, *MAP2K3*, *FRG1B*, *IGSF3*, and *DUSP22*) that showed monoallelic expression across all testable tissues (N≥5) in the RNA-Seq data. Two of these genes were mono-allelically expressed across all ten tissues: NDN, which is a known maternally imprinted gene [18], and MAP2K3, which has known allele-specific expression bias [19]. For all five genes, the same allele was mono-allelically expressed in all testable tissues suggesting that these genes are not imprinted in a tissue-specific manner.

### Patterns of Allele-Specific Expression across Tissues

**Figure 3:**
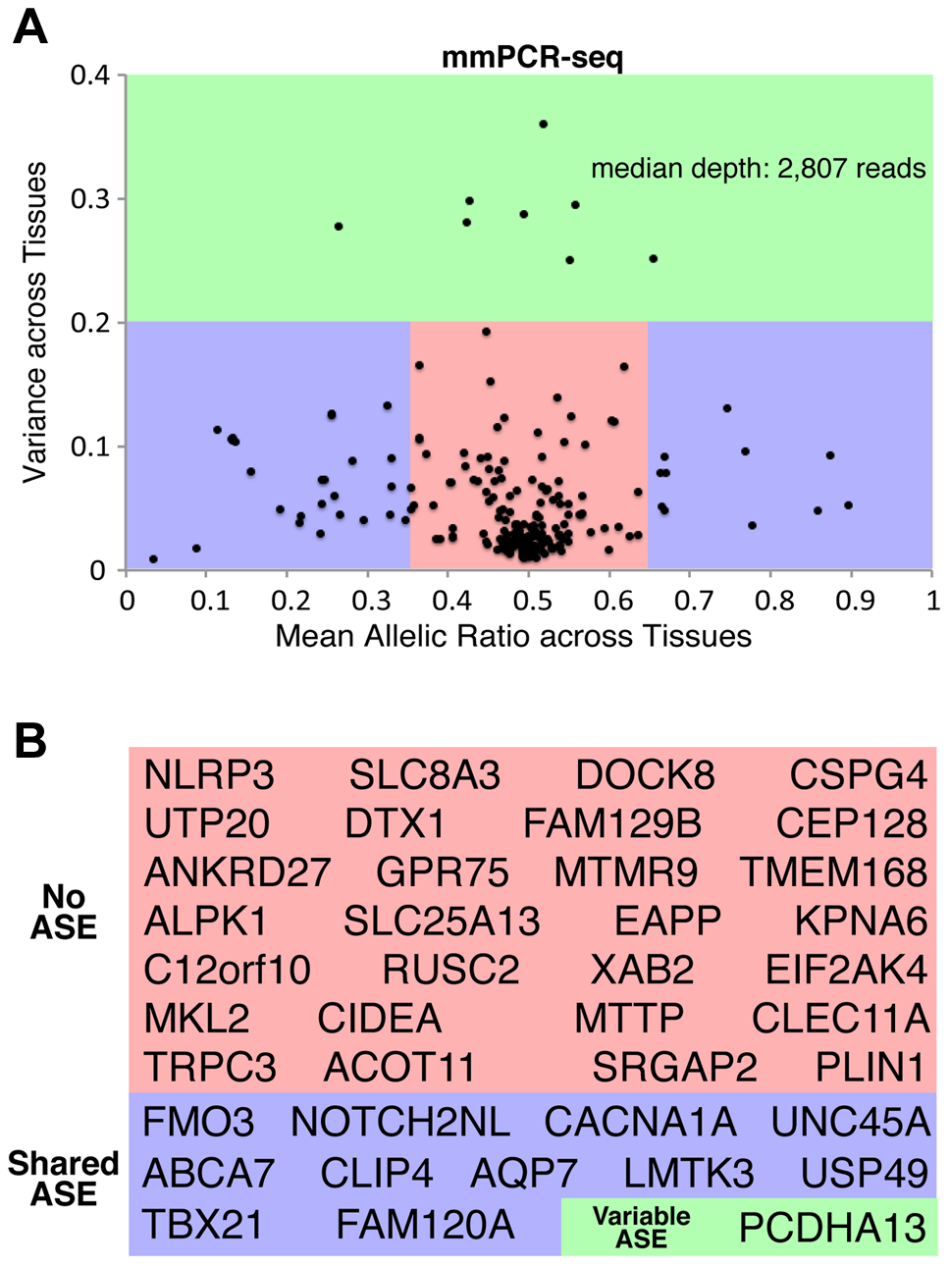
Patterns of ASE across tissues and their influence on rare deleterious variant interpretation. (**A**) The distribution of allelic ratios across tissues indicates that most heterozygous sites have bi-allelic expression across all tissues (no ASE, red). A subset of sites exhibits ASE that is consistent between all tissues (shared ASE, blue). However, a small fraction of sites exhibit ASE that is tissue-specific (variable ASE, green). The mean allelic ratio is on the x-axis and the variance (standard deviation) of the allelic ratio is on the y-axis. Allelic ratios were calculated for all sites tested by mmPCR-Seq. The reproducibility between replicates for the three groups (non-ASE, shared ASE and variable ASE), as well as the classification of non-ASE and ASE (shared ASE plus variable ASE) is is 93.3% and 95.7%, respectively. (**B**) Genes with rare and deleterious nsSNPs were stratified into those that exhibited no ASE (red), shared ASE (blue), and variable ASE (green) across different tissues. The reproducibility of genes classified as shared ASE and variable ASE between replicates is 100%.

The majority of sites tested by mmPCR-Seq have equal expression of both alleles, as expected. However, many sites exhibit consistent or variable allelic patterns across different tissues (Figure S15). By comparing the mean and variance of allelic ratios as quantified through mmPCR-Seq across tissues, we stratified sites into those that exhibited no ASE, shared ASE and variable ASE across tissues. Due to the inherent nature of the binomial test, minor deviations from equal allelic expression will appear significant with high read coverage and therefore p-value significance alone is not sufficient for distinguishing between these classes. Therefore, we also took effect size (allelic ratios) into account when classifying sites as ASE. However, the definition of what constitutes a biologically important allelic effect is not easily discernable; therefore, to distinguish between each group, we accounted for previously reported definitions of ASE [20], [21] and applied cutoffs based on the reproducibility of both the allelic ratio and its variance across replicates (Figure S16). Variants were classified as non-ASE sites if the allelic expression was balanced (mean allelic ratio = 0.5+/−0.15) and if there was low variance (σ^2^<0.2) of the allelic ratios for all tissues tested. Variants were classified as shared ASE sites if they had a significant *p*-value (*p*<0.01), an imbalanced mean allelic ratio (0.35<mean allelic ratio <0.65), and non-variable allelic ratios (σ^2^<0.2) across all tissues. Lastly, variants were classified as variable (tissue-specific) ASE sites if they had a significant *p*-value (*p*<0.01) and variable allelic ratios (σ^2^>0.2) across tissues. The reproducibility of the groups between replicates was tested at varying allelic ratio and variance cut-offs (Figure S16) and was also assessed when the pancreas and frontal lobe, two tissues that had high variability between replicates, were removed (Figure S11). The concordance between replicates increases as the variance cut-off increases and reaches a plateau of ∼95% at a variance of 0.2. Since the greatest reproducibility is observed when the ASE cutoff is <0.35 or >0.65, the variance cutoff is 0.2, and the pancreas is removed, these cut-offs were chosen for Figure 3. Using these cut-offs, the reproducibility between replicates for the three groups (non-ASE, shared ASE and variable ASE) is 93.3%. The reproducibility between replicates for the classification of non-ASE and ASE (shared ASE plus variable ASE) is 95.7%. In total, for sites tested with mmPCR-Seq, 172 showed no ASE across tissues, 52 showed shared ASE, and 8 showed variable ASE (Figure 3A). These proportions are similar to those obtained with RNA-Seq (Figure S17). We then tested if sites exhibiting shared or variable ASE are more likely to be deleterious sites compared to sites exhibiting no ASE. Of the sites exhibiting no ASE, only 25.0% are deleterious. Comparatively, we find no significant enrichment in deleteriousness among sites which exhibit variable ASE compared to non-ASE sites(p = 0.423, Fisher's exact test; not significant); however, a significantly higher proportion of shared ASE sites (42.3%) are deleterious compared to non-ASE sites (p = 0.022; Fisher's exact test).

Next, we investigated the relationship between ASE effect sizes and direction of effect across tissues. Figure S15 highlights the range of effect sizes and directions of effect seen across tissues. By focusing on the range of allelic ratios for variants tested in three or more tissues, we further reviewed the distribution of minimum and maximum allelic ratios observed across all tested tissues (Figure S18). As expected, most sites have an allelic ratio around 0.5, and imbalanced loci show similar direction of effect. Interestingly, several sites exhibit opposing directions of effect in different tissues. For example, heterozygous sites in genes *PCDHA13, SCRIB, and PDE4DIP* have a major flip in direction of effect from an alternate allele ratio less than 0.2 to an alternate allele ratio greater than 0.8 across tissues. Four additional heterozygous sites have a large directional flip from an alternate allele ratio less than 0.4 to greater than 0.8, and five more heterozygous sites have a directional flip from an alternate allele ratio less than 0.2 to greater than 0.6.

To determine if gene expression level informed allelic expression level, we investigated the relationship between gene expression and allelic expression level as measured by mmPCR-Seq (Figure S19). As expected, due to the nature of mmPCR-Seq, no general pattern between absolute expression levels and ASE was observed. Four sites (circled in Figure S19) did have noticeably lower non-reference allele ratios and lower gene expression levels in the pancreas, stomach and lung; however these outliers were not enriched in any variant class and did not influence distinction of variable versus shared ASE.

### Allele-Specific Expression of Rare Deleterious Variants across Tissues

By focusing on patterns of ASE for rare deleterious variants in this individual, we identified 40 sites corresponding to 40 unique genes which were quantified by mmPCR-Seq across three or more tissues. Of these genes 28 exhibited no ASE across tissues, 11 exhibited shared patterns of ASE across tissues and 1 exhibited variable ASE (Figure 3B; Figure S20). We next investigated if genes with different patterns of ASE have relevant disease associations using the Online Mendelian Inheritance in Man (OMIM) database of heritable diseases (Table S4) [22]. Although the OMIM database a limited catalog of genomic variants, OMIM variants serve as examples of the pathogenic consequences of deleterious alleles. Among those that exhibit shared ASE is the *FMO3* gene, which encodes a monooxygenase enzyme responsible for hepatic metabolism and whose deficiency causes the rare Mendelian disorder trimethylaminuria that is manifested in a range of phenotypes (OMIM 602079; Figure S20) [23], [24]. Here, the shared allelic effect is detectable in all tissues, but the strongest effect against the deleterious allele is detected in the liver (non-ref to ref allelic ratio = 0.16; Figure S20). In contrast, no ASE patterns are observed for a deleterious SNP located in the gene encoding a cryopyrin (*NLRP3*), which is associated with the Mendelian disease Muckle-Wells Syndrome (OMIM 191900) and associated with inflammasome function and immune responses. The single deleterious site that demonstrates variable ASE is a gene encoding a protocadherin (*PCDHA13*). In the skeletal muscle and heart, the deleterious allele exhibits greater expression than the normal allele, but in the liver and colon the deleterious alleles exhibits less expression. Of interest, *PCDHA13*, which is known to play a critical role in establishing specific cell-cell connections in the brain, shows no strong patterns of ASE in the two disease-relevant neural tissues, frontal lobe and cerebellum. While the consequences of allelic expression of this individual's deleterious alleles are unknown, different patterns of allelic expression across tissues highlight the potential importance of testing multiple tissues to better elucidate the functional context of rare, deleterious alleles.

### Allele-Specific Expression of Loss-of-Function Variants across Tissues

**Figure 4:**
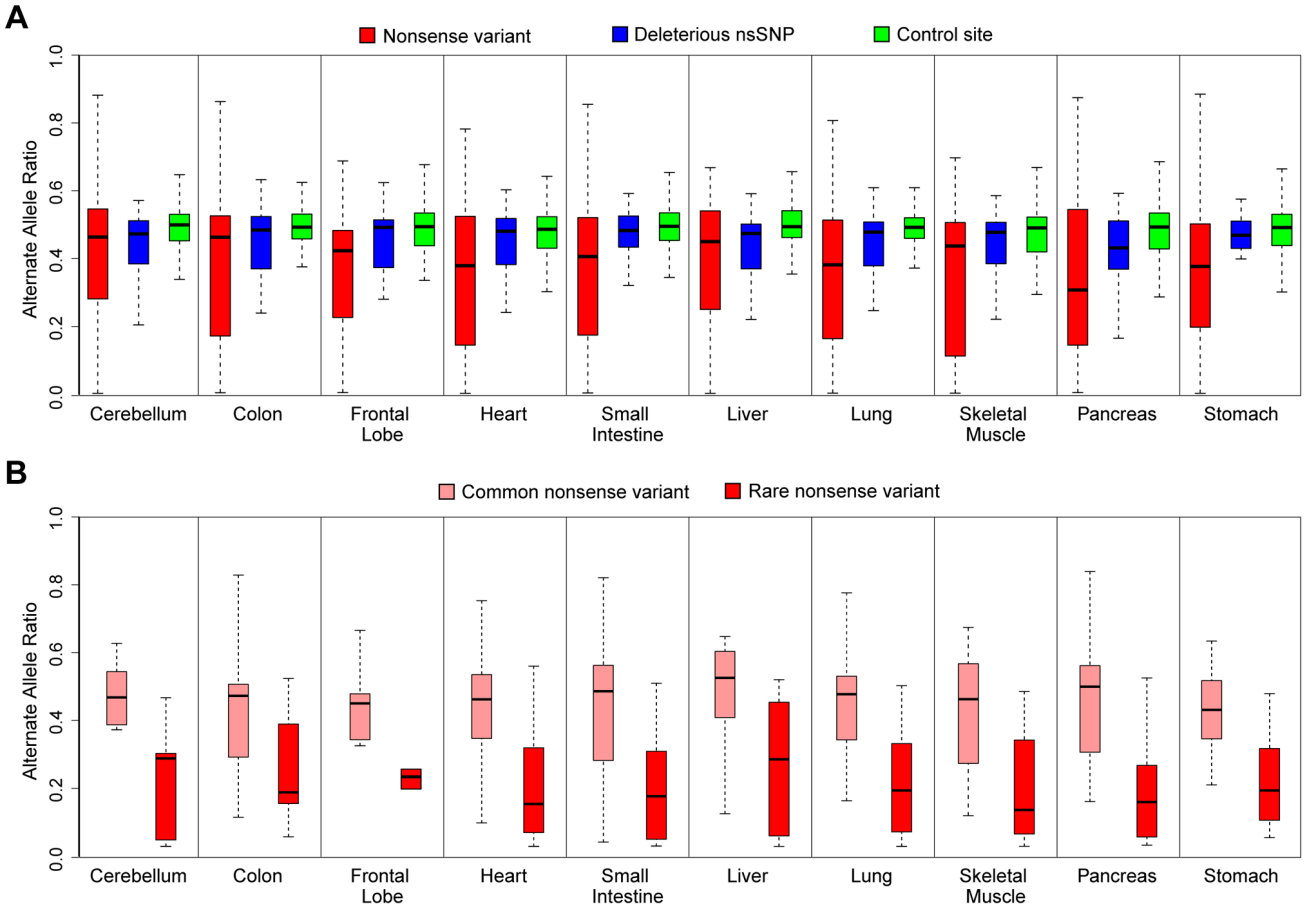
ASE analysis of rare deleterious nsSNPs and nonsense variants by mmPCR-Seq. (**A**) ASE analysis of nonsense variants (red), rare deleterious nsSNPs (blue), and control sites (green) tested by mmPCR-Seq in different tissues. The control sites are random heterozygous sites in the individual's genome. Rare, deleterious nsSNPs and nonsense alleles have significantly reduced expression compared to controls. This observation is most significant for loss-of-function variants where the nonsense allele is likely removed through nonsense-mediated decay (student's t-test, p<0.05, see Table S5). (**B**) ASE analysis of rare (red) and common (pink) nonsense variants tested by mmPCR-Seq data across different tissues. Common nonsense variants are defined as those with a minor allele frequency greater than 5% across the 1000 Genomes population data. Rare nonsense alleles show significantly reduced expression compared to common nonsense alleles (student's t-test, p<0.05).

Loss-of-function alleles that introduce premature stop codons have been identified to exhibit patterns of allelic expression indicating nonsense-mediated decay (NMD) [1]. We sought to test the extent of this impact across different tissues. Indeed, comparison of ASE data using mmPCR-Seq for nonsense (stop-gained) and control sites indicates considerable reduction in the expression of the nonsense allele across all tissues (Figure 4A and Table S5). We also observed lowered expression of rare, deleterious alleles at heterozygous sites compared to control sites (*p*<0.05, student's t-test). This observation has been previously reported in a single cell-type, with a possible explanation for this phenomenon being that lowly-expressed alleles can better tolerate the fitness impact of deleterious protein-coding alleles [25], [26]. Furthermore, we identified that rare (MAF<5%) nonsense alleles exhibited even stronger evidence of nonsense-mediated decay than common alleles (Figure 4B). To ensure that genotype errors and mappability did not affect this observation, we compared RNA allelic bias to DNA allelic bias from exome-sequencing. Nonsense variants were removed from the analysis if the alternative allelic ratio was below 0.2 in both tissues. This filtration step ensures that genotyping and mappability of non-reference variants did not influence our observation that rare nonsense variants have decreased allelic expression compared to common nonsense variants. This observation suggests that haplotypes that harbor rare nonsense variants are either considerably unlikely to be expressed or altered transcripts are being efficiently degraded by the NMD machinery.

In conclusion, despite the feasibility of sequencing individual genomes, the functional impact of potentially pathogenic protein-coding variants remains difficult to ascertain by DNA sequencing or computational prediction methods alone. The incorporation of transcriptome data can enhance the interpretation of such variants by providing insight into their patterns of ASE. We demonstrate the advantage of ASE for interpretation of pathogenic protein-coding allele by generated high resolution measurements of ASE for these variants across multiple tissues. Such data enables us to identify the extent to which these alleles are modified by regulatory effects and the extent to which this effect is detectable across tissues. We highlight as many as a 1/3 of all deleterious alleles are imbalanced and that nonsense alleles show characteristic and consistently lower expression across multiple tissues. Ultimately, by coupling interpretation of personal genomes with their corresponding transcriptomes, these results highlight that it may be possible to better understand the impact of pathogenic protein-coding variants within different tissues of an individual.

## Materials and Methods

### Collection of Tissue Samples

In order to investigate the differential allelic effects of divergent tissues in a single individual, we obtained the genomic DNA and RNA for ten somatic tissues (cerebellum, frontal lobe, pancreas, stomach, small intestine, colon, heart, lungs, liver, and skeletal muscle) from Biochain Institute, Inc (Newark, CA, USA). The samples were collected post-mortem from a healthy 25-year-old male with no significant medical history.

### Whole Exome Sequencing

Genomic DNA from the frontal lobe and small intestine were prepared for exome sequencing. The enrichment of targeted regions (consensus coding sequence definition of exons and flanking introns, ∼50 Mb) was performed using the Agilent SureSelect Human All Exon 50 Mb Kit (Agilent Technologies, Santa Clara, CA, USA) following the manufacturer's recommended protocol. Paired-end libraries were constructed using the Illumina Paired End Sample Prep Kit following the manufacturer's instructions and sequencing was carried out using the Illumina HiSeq 2000 platform (Illumina, San Diego, CA, USA). Exome sequence data was processed through a pipeline based on Picard (http://picard.sourceforge.net/) with base quality score recalibration and local realignment at known indels and BWA [27], for mapping reads to the human reference genome (build hg19). GATK version v2.3-13 [28] was used for SNP calling, with the default filters, and the additional parameters: -T UnifiedGenotyper; –downsample_to_coverage 75; –genotype_likelihoods_model BOTH; -contamination 0.0; -nct 1. For ASE detection (described below), we filtered for heterozygous variants that were present in both the frontal lobe and small intestine.

### Whole Transcriptome Sequencing

Paired-end RNA-Seq libraries were prepared using the Illumina TruSeq RNA Sample Preparation kit. PolyA+ RNA was isolated using Sera-Mag oligo(dT) beads (Thermo) and fragmented with the Ambion Fragmentation Reagents kit. Complementary DNA (cDNA) synthesis, end repair, A-base addition and ligation of the Illumina-indexed adaptors were performed according to Illumina's protocol. Each sample was barcoded and all samples were sequenced on one lane of the Illumina HiSeq 2000 platform (2×100-nt read length). In total, we obtained 13.3±3.7 (mean ± SD) million paired end reads per sample. We assessed the sequence quality using the publicly available software FastQC. For each sample, we examined per-base quality scores across the length of the reads to ensure that >95% of the reads had >Q60 for bases 1–100. Reads were mapped by TopHat (version 2.0.0) to the known transcriptome (-G option; Gencode version 7 annotations) the human reference genome (hg19) using default parameters [29]. Cufflinks (version 2.0.2) was used to quantify gene expression for known transcripts (-G option; Gencode version 7 annotations) using the default parameters [30].

### Targeted Allelic Sequencing by mmPCR-Seq

To quantify allele-specific expression at lowly expressed site, we applied a high-throughput method that couples microfluidics-based multiplex PCR and deep sequencing (mmPCR-Seq) [11]. We designed primers and applied this technique to 74 deleterious nonsynonymous variants, 50 nonsense variants, and 205 control variants. The control sites are common (MAF>0.05), non-deleterious variants. First, multiplexed PCR reactions were carried out using the Fluidigm Access Array for each sample. Then, the PCR products were indexed using barcoded adaptor primers via a single PCR reaction for each tissue sample. All indexed samples were pooled and purified using a Qiagen RNeasy. Six picomoles were loaded into one lane of an Illumina MiSeq for deep sequencing. The sequence reads were mapped to the human reference genome (hg19) using the Spliced Transcripts Alignment to a Reference (STAR, version 3.2) aligner [31]. Since we targeted together specific heterozygous sites in the genome, the default parameters were modified (minimum score and match filters lowered from 0.66 to 0.3) to increase the number of mapped reads.

### Allele-Specific Expression

Allele-specific expression was determined on a per-heterozygote-site per-tissue basis using the pipeline depicted in Figure S4 and available online (http://montgomerylab.stanford.edu/resources.html). First, mapped reads were sorted using the Samtools (version 0.1.7) [32]. Next, Samtools mpileup was used to call variants from the aligned reads using a list of known heterozygous sites from the individual. Heterozygous sites with a base quality score (MAQ) below 10, individual allele read depth below 5 and a total (both alleles) read depth below 20 were filtered out. Next, we calculated the reference to non-reference allele mapping ratio for each tissue. To test for ASE, we performed a binomial statistical test for each heterozygous site in each tissue modifying *p* to be the empirical probability of observing a reference versus non-reference allele across all sites. A significance cut-off of 0.05 and 0.01 were used for the RNA-Seq and mmPCR-Seq data, respectively.

### Data Access

The raw mmPCR-seq data has been submitted to the NCBI Gene Expression Omnibus (GEO) (http://www.ncbi.nlm.nih.gov/geo/) under accession number GSE51769. The code for ASE detection pipeline can be found online (http://montgomerylab.stanford.edu/resources.html).

## Supporting Information

Figure S1 Base quality distribution for RNA-Seq reads. The base quality distribution for the 100-bp paired-end RNA-Seq reads from the Illumina Hi-Seq 2000 platform. The mean quality score at each base position for each tissue sample is plotted for read 1 (left) and read 2 (right). The y-axis is the average quality value, the x-axis is the base position, and each colored line represents a corresponding tissue sample as indicated by the legend (far right).
(TIF)

Figure S2 Mapping RNA-Seq reads. For all tissues except the stomach, ∼90% of the reads mapped uniquely to the human genome. Reads under 20 bp were unmapped and reads that mapped to multiple regions of the genome (multi-mapping reads) were discarded for future analysis.
(TIF)

Figure S3 Reference mapping bias distributed by base quality scores. The reference to non-reference mapping bias for each tissue exhibits no distinct patterns with respect to specific tissue sample or base quality scores.
(TIF)

Figure S4 Pipeline for the detection of allele-specific expression.
(TIF)

Figure S5 Distribution of allele-specific expression for RNA-Seq. Density plots illustrate the distribution of the alternate allele ratio for each tissue for all heterozygous sites that are expressed. The alternate allele ratio was calculated from RNA-Seq reads as the fraction of alternate allele reads divided by the total reads. In ASE analyses using RNA-Seq reads, it is important to evaluate if mapping bias exists that results in the favoring of reads harboring the reference allele at heterozygous sites. In the absence of mapping bias, the average allelic ratio is expected to be 0.5, assuming that ASE is exhibiting in a minor fraction of heterozygous sites.
(TIF)

Figure S6 Quality control filtering of ASE sites. The identification of ASE sites from RNA-Seq data required quality control filter to identify high-confident sites. The x-axis shows the reference to non-reference mapping ratio for each sample and the y-axis shows the percentage of ASE sites remaining after each quality-control filter. The base quality and read depth filters resulted in a modest (∼10%) reduction in ASE sites. The p-value (p<0.05), bi-allelic expression, and intragenic location filters removed over 50% of the sites for each tissue. The proportion of sites removed after each filter shows no correlation with the reference to non-reference mapping bias for the RNA-Seq samples.
(TIF)

Figure S7 Selection of LoF sites for mmPCR-Seq testing. Rare and deleterious nonsynonymous SNPs were selected for testing by mmPCR-Seq. Rare and deleterious nsSNPs are defined as SNPs not observed in dbSNP, 1000Genomes, or ESP, and annotated as damaging and deleterious by SIFT and POLYPHEN. The nonsense variants selected for testing were identified as variants that affect every full transcript in the gene.
(TIF)

Figure S8 mmPCR-Seq reads by tissue. Two technical replicates of mmPCR-Seq were performed for each tissue. Since we have observed very high concordance of allelic ratios between technical replicates using mmPCR-Seq, the reads from each replicate were merged.
(TIF)

Figure S9 Mapping mmPCR-Seq reads. The total reads generated per tissue from the mmPCR-Seq experiments were mapped to the reference genome using the STAR aligner. For every tissue sample, approximately 98% of the reads mapped uniquely to the reference genome.
(TIF)

Figure S10 Correlation of effect size for mmPCR-Seq technical replicates. Two technical replicates of mmPCR-Seq were performed and the ASE effect size was quantified. For each tissue, the effect size for each technical replicate was plotted to demonstrate the correlation between technical replicates for mmPCR-Seq.
(TIF)

Figure S11 Variance of effect size for mmPCR-Seq technical replicates. The absolute difference in effect size (allelic ratio) between the two replicates for each tissue is plotted at varying read depth. At higher read depths, there is less variability between replicates. However, even at low read depths (<200), the variability is low for most tissues, except for the pancreas and frontal lobe, which are known to have low RNA quality post-mortem.
(TIF)

Figure S12 Comparison of coverage of LoF variants using different technologies. (**A**) Comparison of read depth at heterozygous variants using RNA-Seq and mmPCR-Seq data. The tested heterozygous sites have consistently deeper coverage using mmPCR-Seq. (**B**) Comparison of ASE detection using RNA-Seq and mmPCR-Seq data. The comparison of p-values obtained from the ASE binomial test for matching heterozygous sites indicates increased enrichment for significant ASE effects using mmPCR-Seq.
(TIF)

Figure S13 Distribution of alternate allele ratio and corresponding p-value for sites tested by mmPCR-Seq.
(TIF)

Figure S14 Pairwise correlation of expression and allelic effect for similar tissues. The Spearman correlation coefficient was determined for the pairwise comparisons of neural tissues (frontal lobe and cerebellum) and intestinal tissues (small intestine and colon) for both expression and allelic effect. Independent of read depth, the correlation of expression for tissues of similar embryonic origin are consistently greater than the correlation of allelic effect.
(TIF)

Figure S15 Distribution of alternate allele ratio across tissues from mmPCR-Seq. The alternate allele ratio (alternate allele reads divided by total reads) was calculated for each heterozygous site tested by mmPCR-Seq in all tissues. As expected, the majority of heterozygous sites have an alternate allele ratio of 0.5. For sites with ASE, there appears to be an equal distribution of expression bias towards both the alternate allele (upper left tail) and reference allele (lower right tail). Interestingly, we observed that some sites had measurably varied alternate allele ratios across tissues, while other tested sites had a consistent alternate allele ratio across tissues.
(TIF)

Figure S16 Reproducibility of ASE groups for mmPCR-Seq technical replicates. The reproducibility of the groups (shared ASE, variable ASE, and no ASE) depicted in Figure 3 between replicates was assessed at varying cut-offs. The correlation between replicates was evaluated at two ASE cut-offs (0.4–0.6 and 0.35–0.65) and at eight variance cut-offs (0.05–0.3) for all tissues as well as without the pancreas and frontal lobe. The concordance between replicates increases as the variance cut-off increases and reaches a plateau of ∼95% at a variance of 0.2. The greatest reproducibility is observed when the ASE cutoff is ASE<0.35 or ASE>0.65, the variance cutoff is 0.2, and the pancreas is removed. Using these cut-offs, the reproducibility between replicates for the three groups (non-ASE, shared ASE and variable ASE) is 93.3%. The reproducibility between replicates for the classification of non-ASE and ASE (shared ASE plus variable ASE) is 95.7%.
(TIF)

Figure S17 Distribution of shared and variable ASE for RNA-Seq data. The distribution of mean values and standard deviations of the allelic ratios across tissues from the RNA-Seq data. Genomic loci with no ASE and low variance (red), ASE and low variance (blue), and ASE and high variance (green) were divided into three gene groups: no ASE, shared ASE, and variable ASE, respectively. The proportion of sites falling into each ASE group is similar to that found from the mmPCR-Seq data.
(TIF)

Figure S18 Distribution of effect size and direction of effect for ASE across different tissues. The minimum and maximum alternate allele ratio observed in any tissue for each mmPCR-Seq site tested in at least three tissues are plotted to demonstrate the range of allelic effects observed across tissues.
(TIF)

Figure S19 Relationship of ASE effect size and gene expression. The relationship between ASE effect size (measured by mmPCR-Seq) and gene expression level (measured by RNA-Seq) across all tissues was examined. There is no correlation between allelic effect size and gene expression level. Four lowly expressed sites had low allelic ratios (circled) but were not enriched in any class of variants or influenced calling of variable ASE within the study.
(TIF)

Figure S20 Examples of genes with deleterious nsSNPs exhibiting shared, variable, and no ASE. The gene *FMO3*, which is associated with the rare Mendelian disorder trimethylaminuria (OMIM 602079), exhibits decreased expression of the deleterious allele across tissues. In contrast, gene *NLRP3*, which is associated with the Mendelian disease Muckle-Wells Syndrome (OMIM 191900), exhibited no ASE across tissues. The gene *PCDHA13*, which encodes a protocadherin, is an example of a gene with variable ASE across tissues; the deleterious allele is underexpressed in certain tissues and overexpressed in other tissues.
(TIF)

Table S1 Identification of high-confidence heterozygous and homozygous variants.
(PDF)

Table S2 Identification of high-confidence common and rare variants.
(PDF)

Table S3 Identification of high-confident RNA-Seq ASE sites by quality-control filtering. To identify high-confident ASE sites, we implemented several quality control filters. We only kept ASE sites which met the following criteria: 1) base quality (BQ) greater than 10; 2) minimum sequencing depth of 10 reads; 3) calculated p-value less than 0.05 from the binomial test; 4) bi-allelic expression; and 5) intragenic location.
(PDF)

Table S4 OMIM genes associated with Mendelian disease phenotypes harboring rare deleterious nsSNP variants.
(PDF)

Table S5 Allelic imbalance of deleterious nsSNPs, nonsense variants, and control sites across tissues.
(PDF)
