# Supplementary figures and images for: Allelic Expression of Deleterious Protein-Coding Variants across Human Tissues
Source: PLoS Genet. 2014 May 1;10(5):e1004304. doi: 10.1371/journal.pgen.1004304 (PMC4006732; doi:10.1371/journal.pgen.1004304)

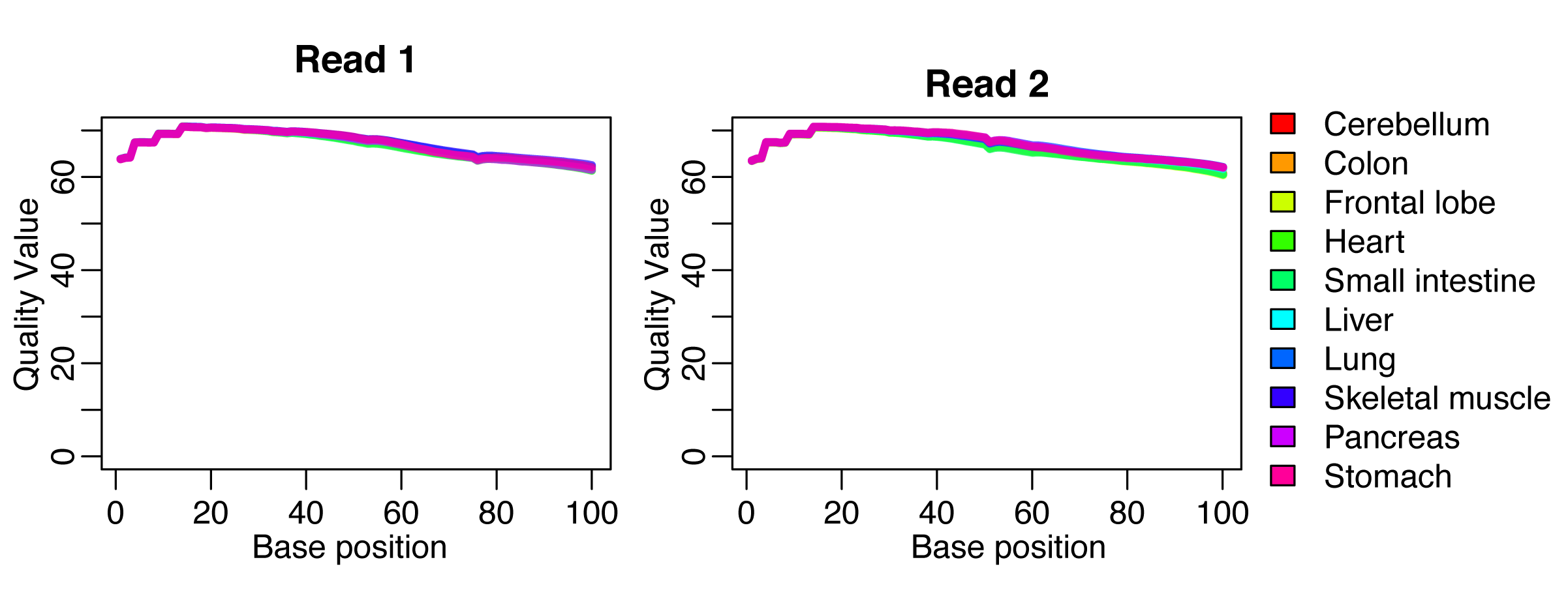

Supplement: Figure S1 — Base quality distribution for RNA-Seq reads. The base quality distribution for the 100-bp paired-end RNA-Seq reads from the Illumina Hi-Seq 2000 platform. The mean quality score at each base position for each tissue sample is plotted for read 1 (left) and read 2 (right). The y-axis is the average quality value, the x-axis is the base position, and each colored line represents a corresponding tissue sample as indicated by the legend (far right). (TIF) [file pgen.1004304.s001.tif]

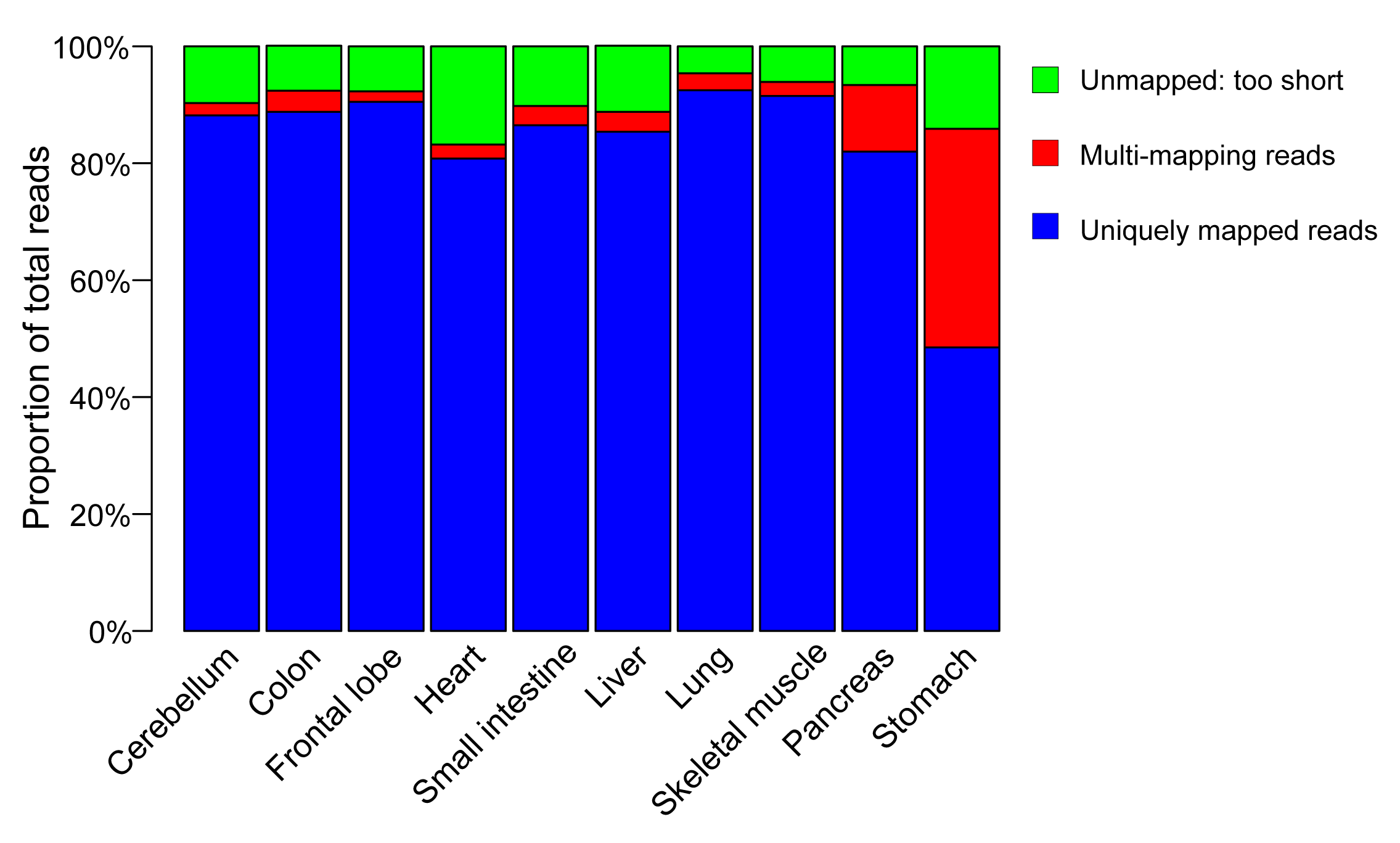

Supplement: Figure S2 — Mapping RNA-Seq reads. For all tissues except the stomach, ∼90% of the reads mapped uniquely to the human genome. Reads under 20 bp were unmapped and reads that mapped to multiple regions of the genome (multi-mapping reads) were discarded for future analysis. (TIF) [file pgen.1004304.s002.tif]

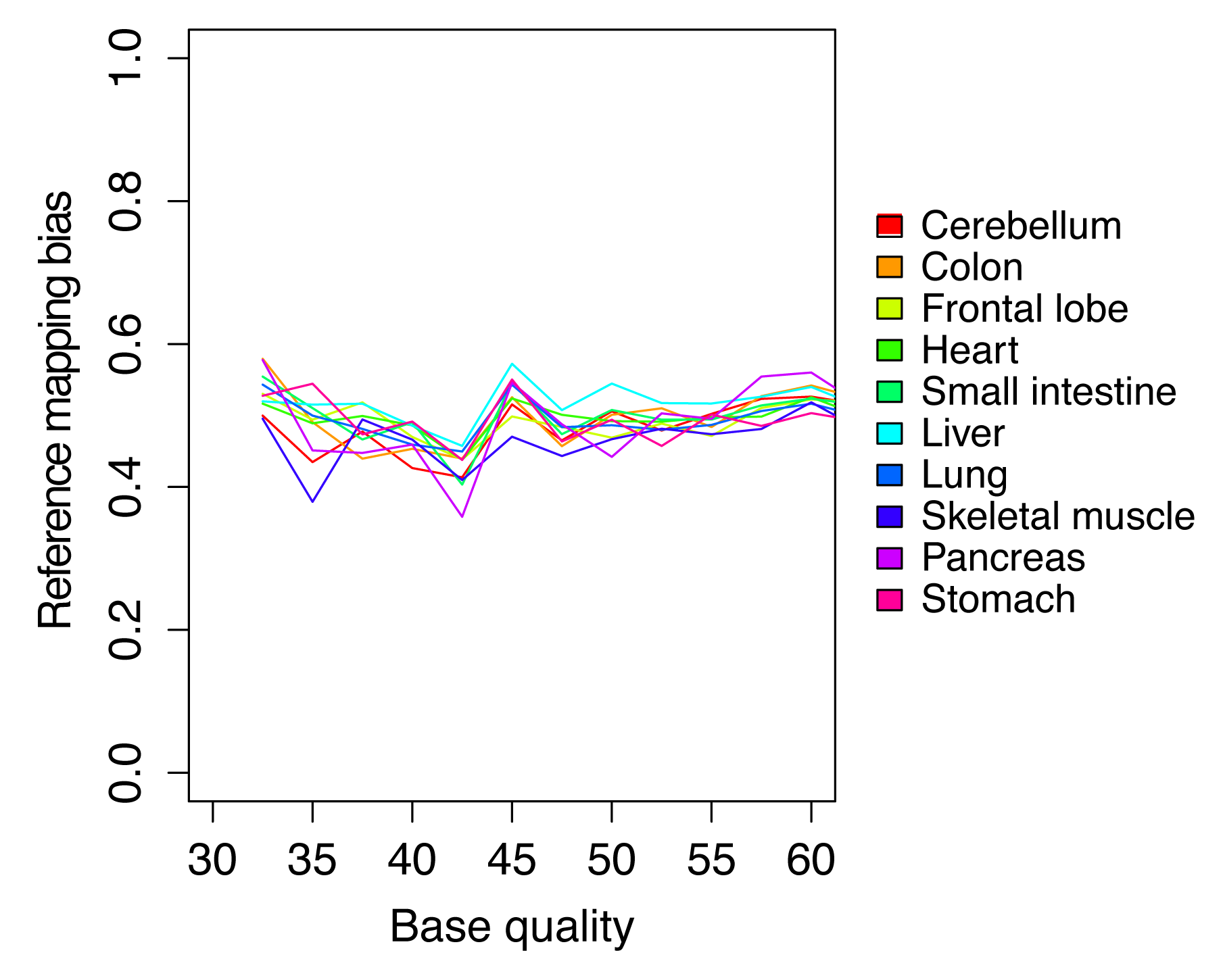

Supplement: Figure S3 — Reference mapping bias distributed by base quality scores. The reference to non-reference mapping bias for each tissue exhibits no distinct patterns with respect to specific tissue sample or base quality scores. (TIF) [file pgen.1004304.s003.tif]

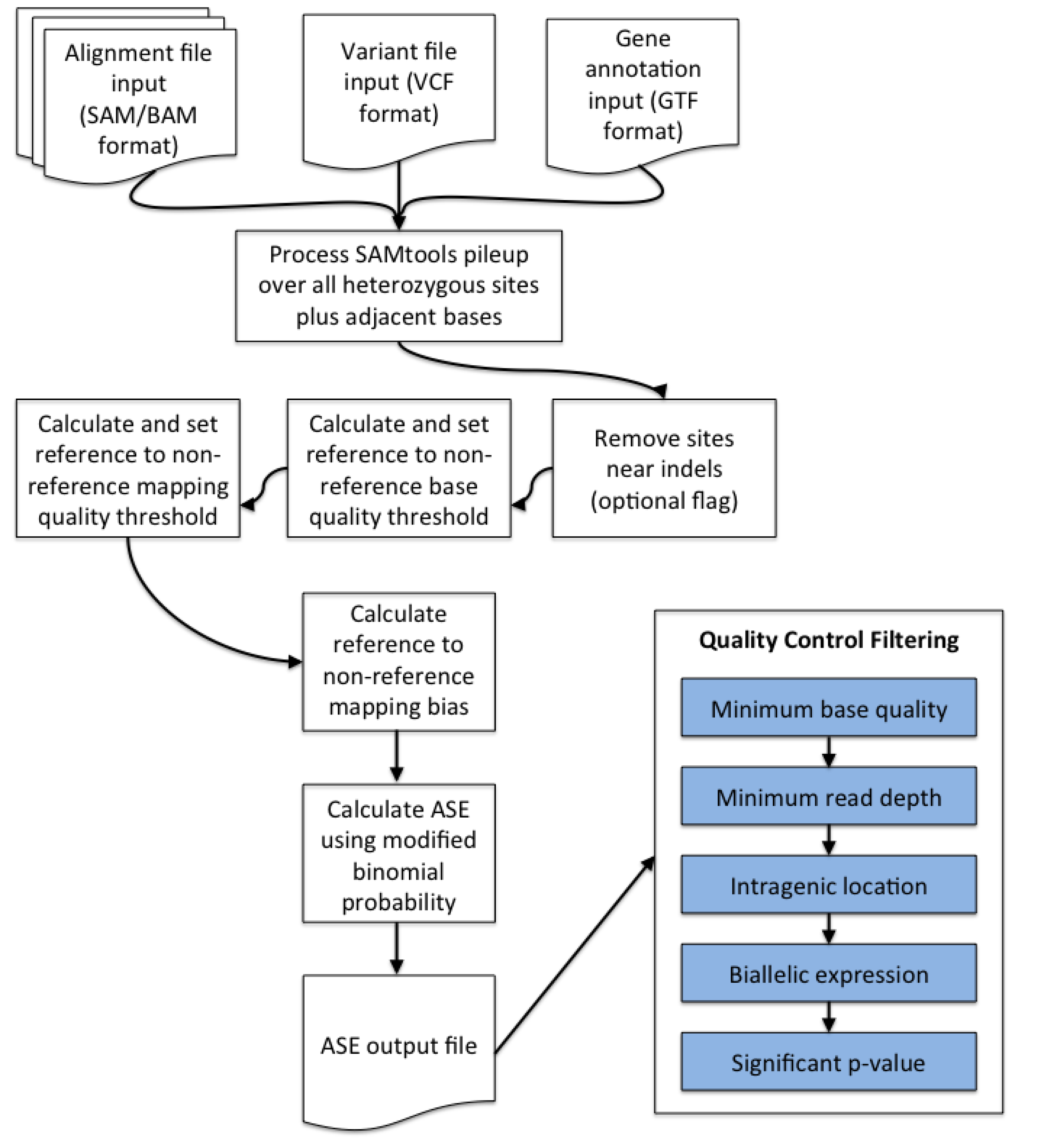

Supplement: Figure S4 — Pipeline for the detection of allele-specific expression. (TIF) [file pgen.1004304.s004.tif]

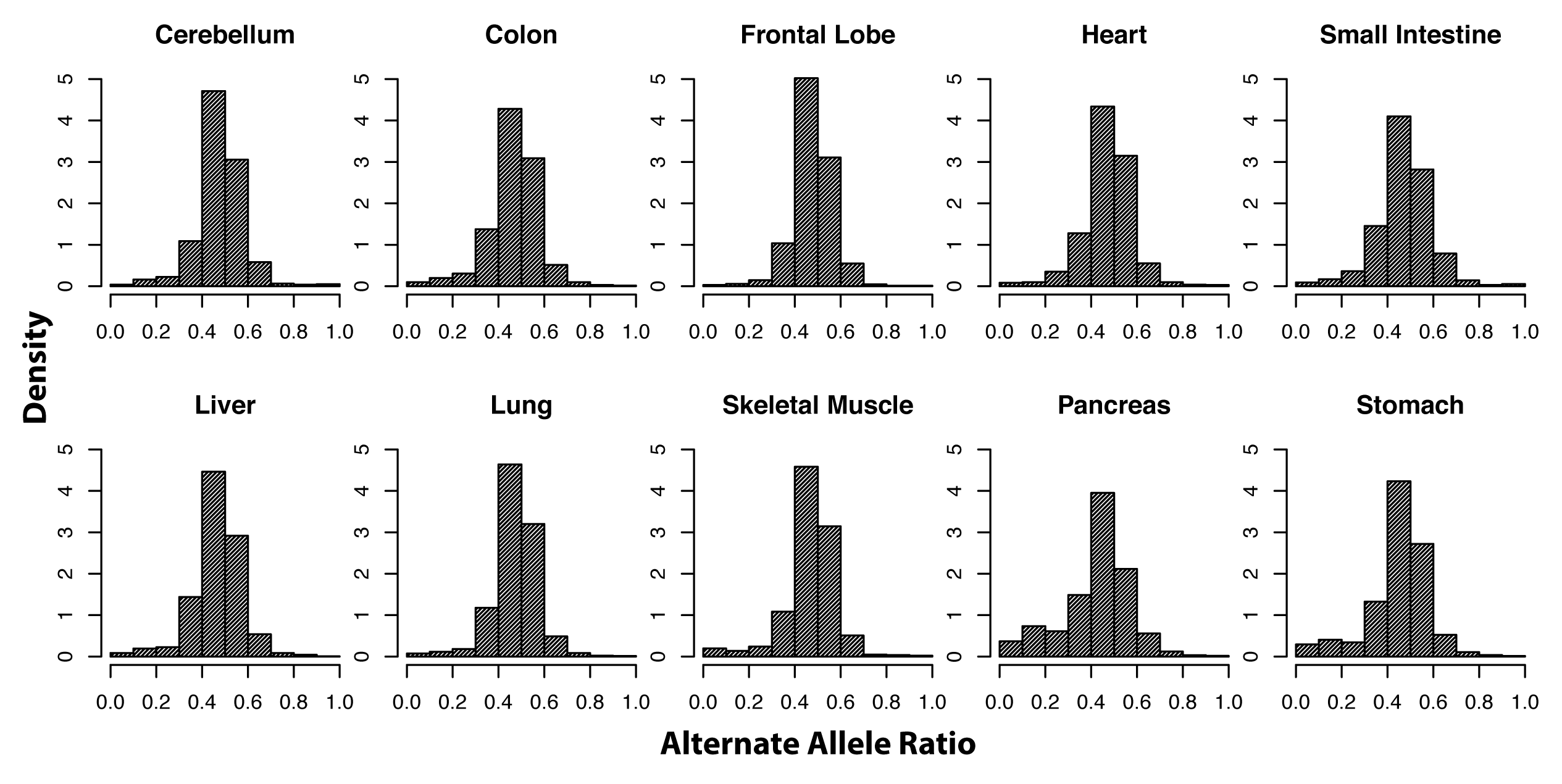

Supplement: Figure S5 — Distribution of allele-specific expression for RNA-Seq. Density plots illustrate the distribution of the alternate allele ratio for each tissue for all heterozygous sites that are expressed. The alternate allele ratio was calculated from RNA-Seq reads as the fraction of alternate allele reads divided by the total reads. In ASE analyses using RNA-Seq reads, it is important to evaluate if mapping bias exists that results in the favoring of reads harboring the reference allele at heterozygous sites. In the absence of mapping bias, the average allelic ratio is expected to be 0.5, assuming that ASE is exhibiting in a minor fraction of heterozygous sites. (TIF) [file pgen.1004304.s005.tif]

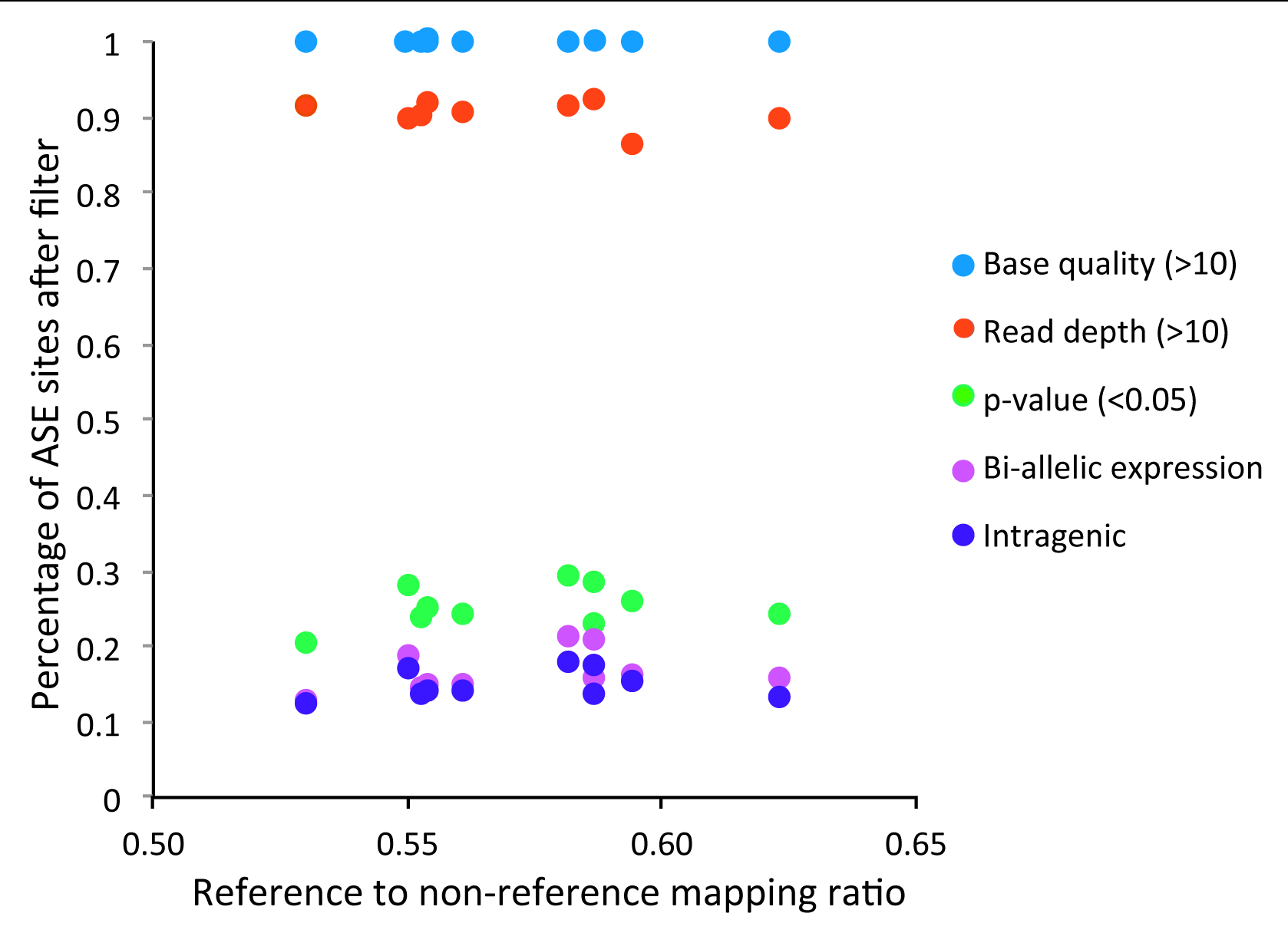

Supplement: Figure S6 — Quality control filtering of ASE sites. The identification of ASE sites from RNA-Seq data required quality control filter to identify high-confident sites. The x-axis shows the reference to non-reference mapping ratio for each sample and the y-axis shows the percentage of ASE sites remaining after each quality-control filter. The base quality and read depth filters resulted in a modest (∼10%) reduction in ASE sites. The p-value (p<0.05), bi-allelic expression, and intragenic location filters removed over 50% of the sites for each tissue. The proportion of sites removed after each filter shows no correlation with the reference to non-reference mapping bias for the RNA-Seq samples. (TIF) [file pgen.1004304.s006.tif]

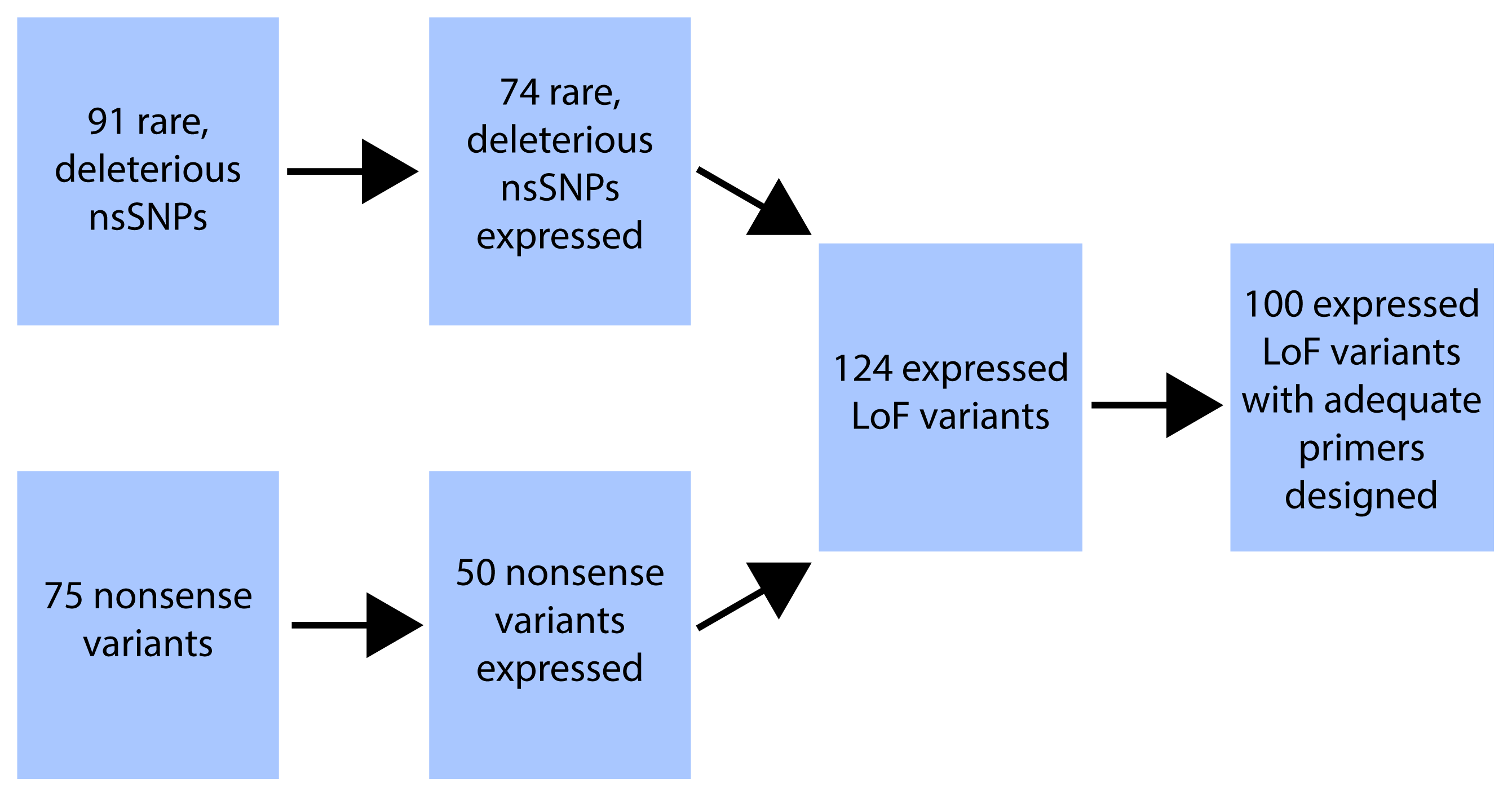

Supplement: Figure S7 — Selection of LoF sites for mmPCR-Seq testing. Rare and deleterious nonsynonymous SNPs were selected for testing by mmPCR-Seq. Rare and deleterious nsSNPs are defined as SNPs not observed in dbSNP, 1000Genomes, or ESP, and annotated as damaging and deleterious by SIFT and POLYPHEN. The nonsense variants selected for testing were identified as variants that affect every full transcript in the gene. (TIF) [file pgen.1004304.s007.tif]

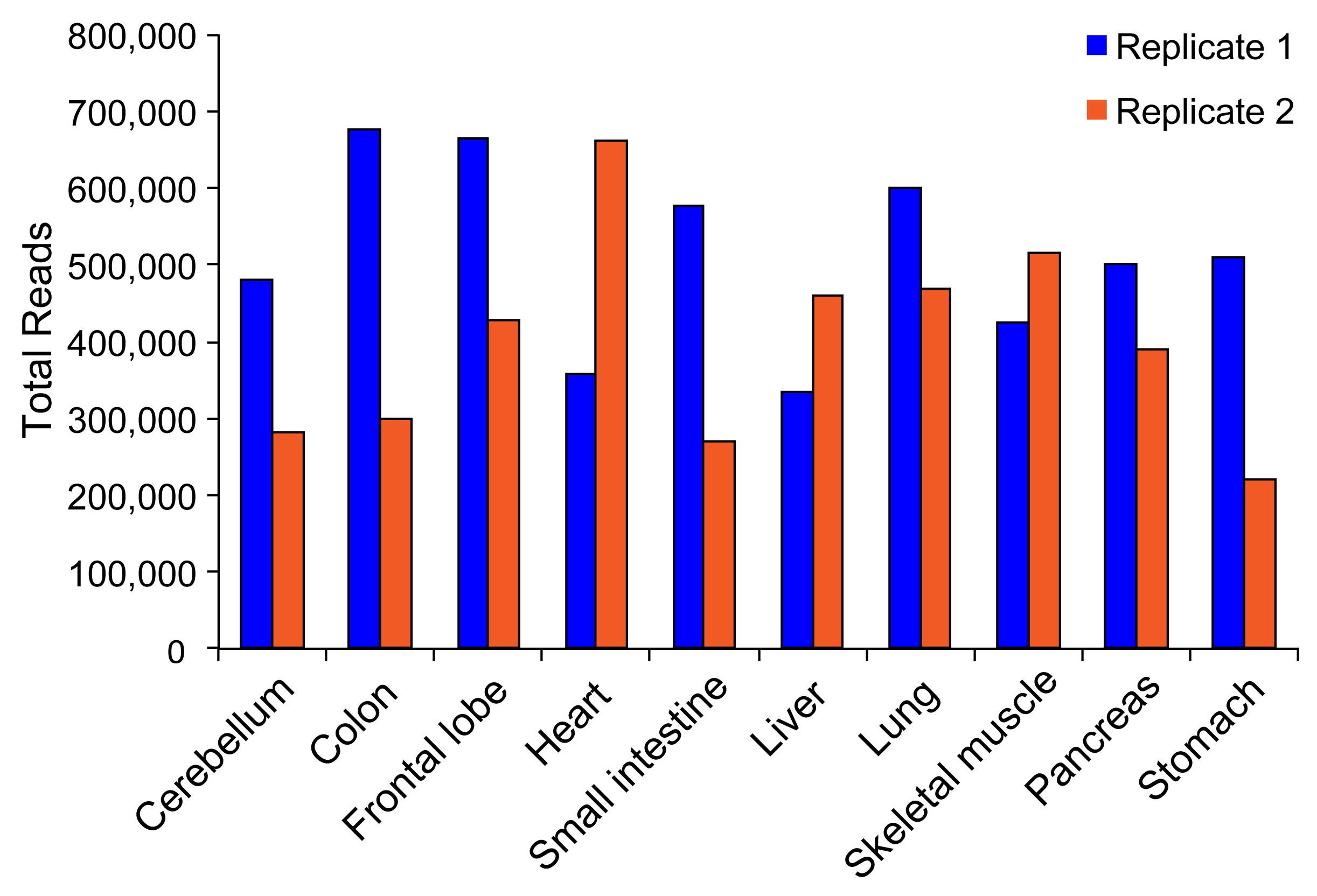

Supplement: Figure S8 — mmPCR-Seq reads by tissue. Two technical replicates of mmPCR-Seq were performed for each tissue. Since we have observed very high concordance of allelic ratios between technical replicates using mmPCR-Seq, the reads from each replicate were merged. (TIF) [file pgen.1004304.s008.tif]

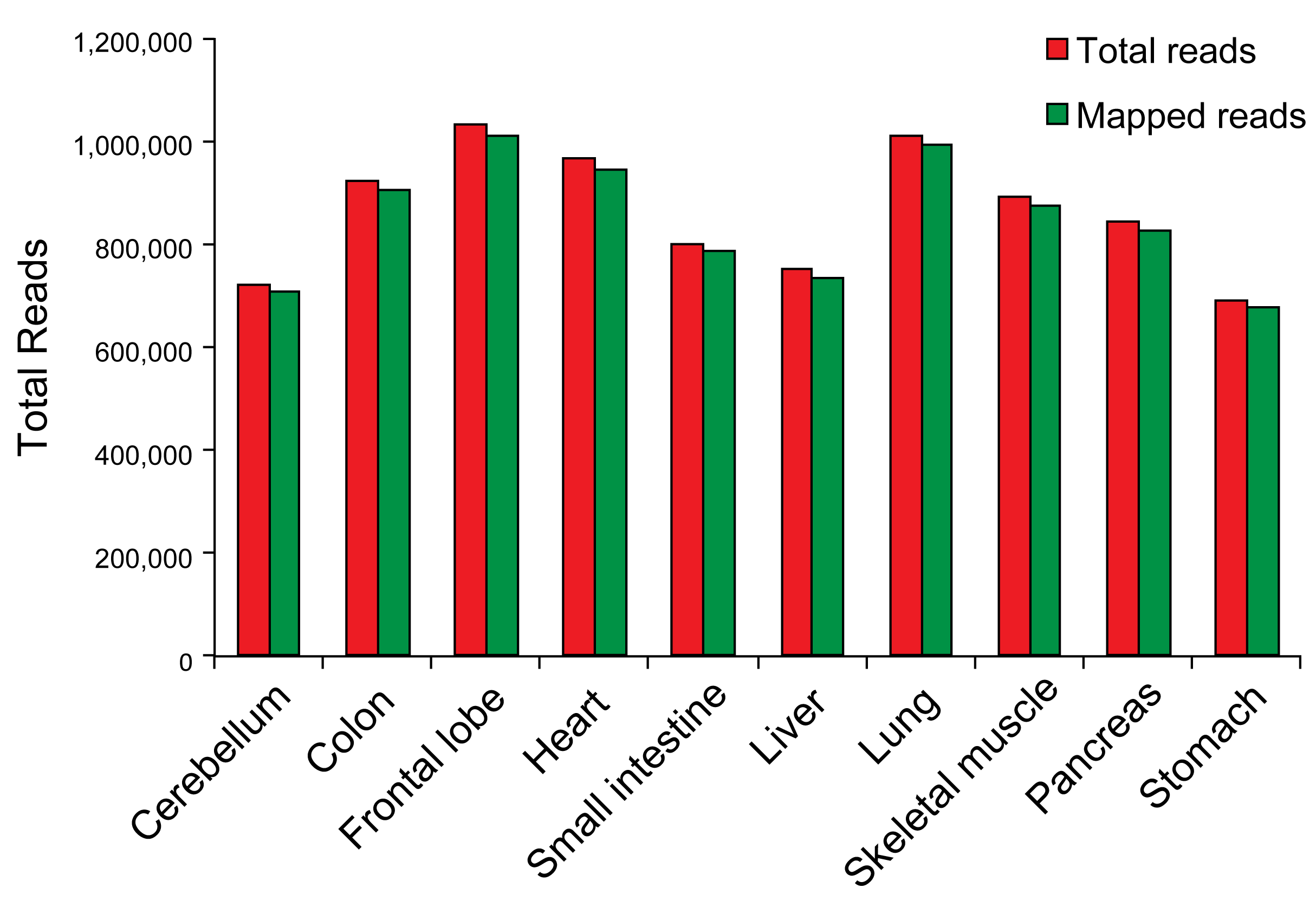

Supplement: Figure S9 — Mapping mmPCR-Seq reads. The total reads generated per tissue from the mmPCR-Seq experiments were mapped to the reference genome using the STAR aligner. For every tissue sample, approximately 98% of the reads mapped uniquely to the reference genome. (TIF) [file pgen.1004304.s009.tif]

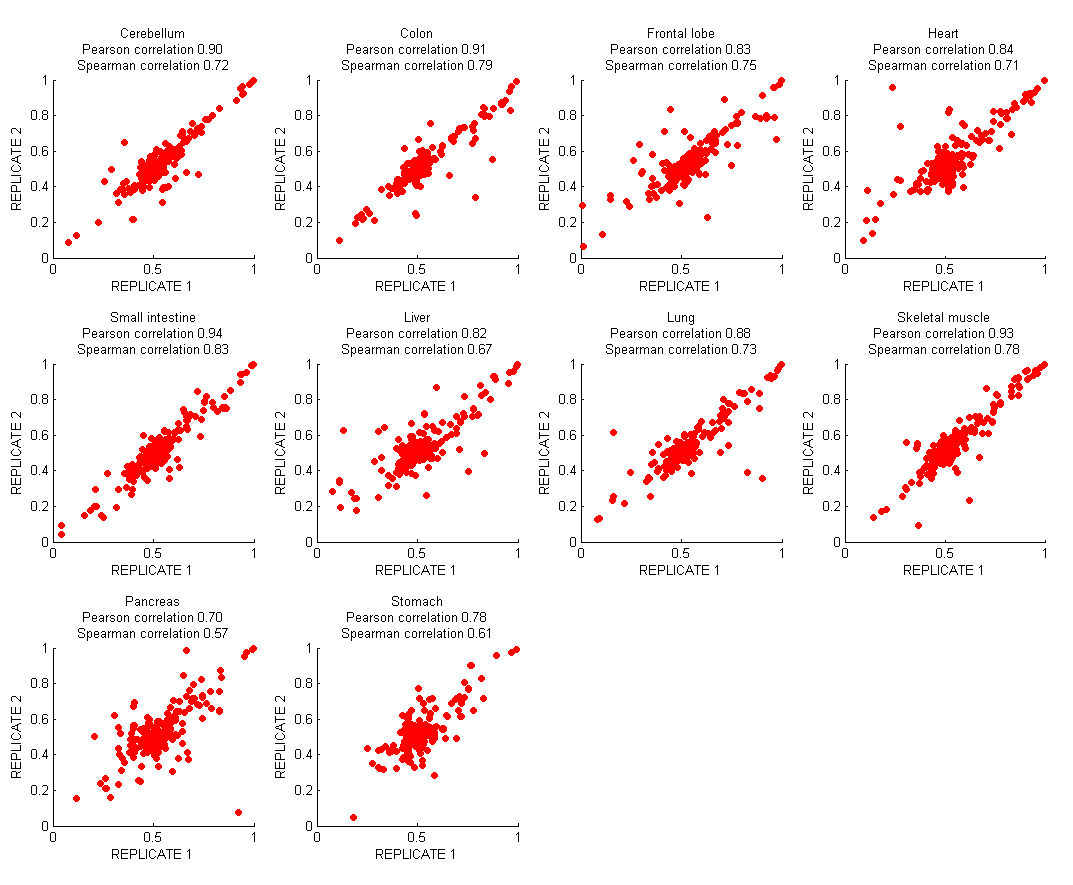

Supplement: Figure S10 — Correlation of effect size for mmPCR-Seq technical replicates. Two technical replicates of mmPCR-Seq were performed and the ASE effect size was quantified. For each tissue, the effect size for each technical replicate was plotted to demonstrate the correlation between technical replicates for mmPCR-Seq. (TIF) [file pgen.1004304.s010.tif]

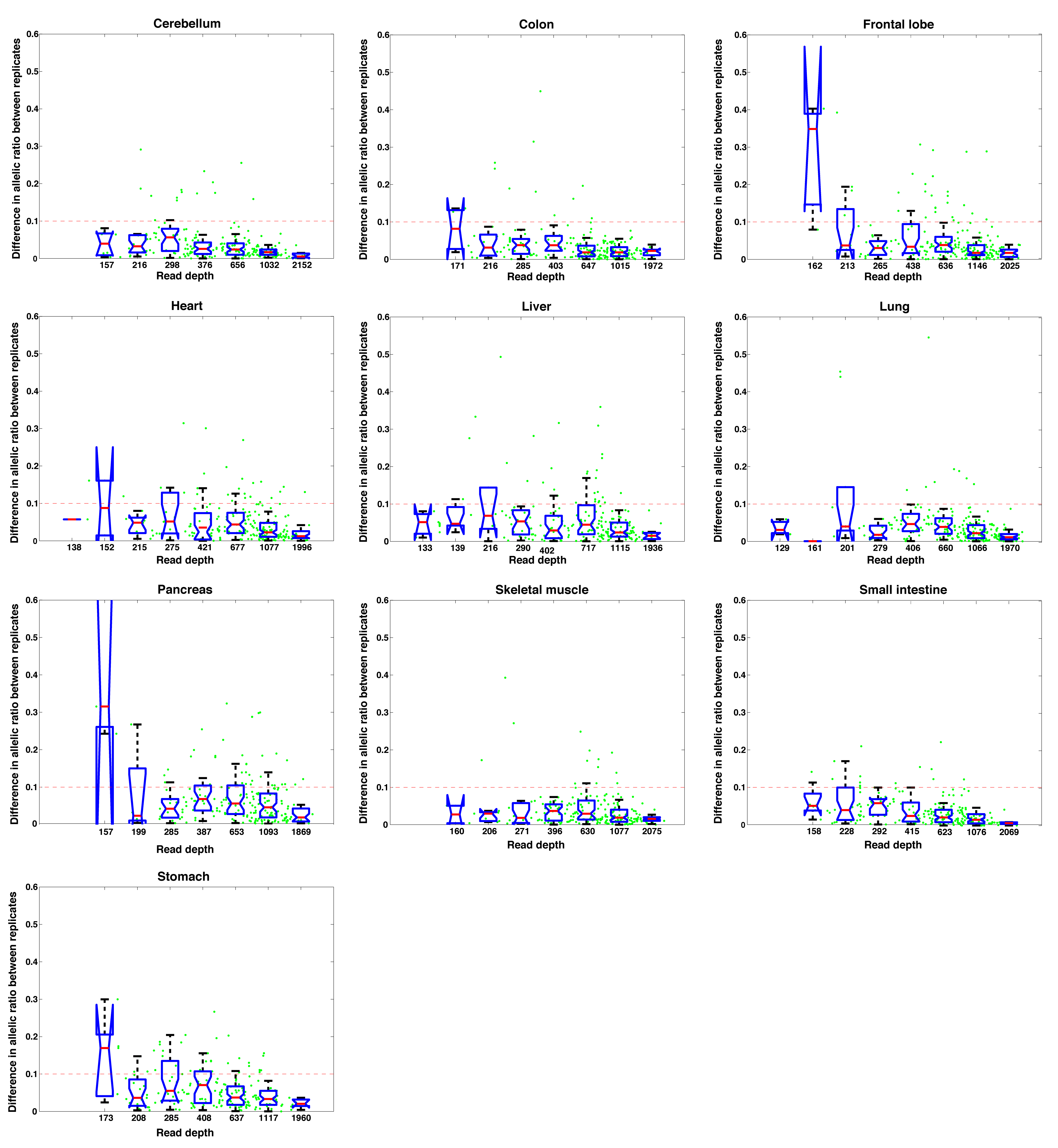

Supplement: Figure S11 — Variance of effect size for mmPCR-Seq technical replicates. The absolute difference in effect size (allelic ratio) between the two replicates for each tissue is plotted at varying read depth. At higher read depths, there is less variability between replicates. However, even at low read depths (<200), the variability is low for most tissues, except for the pancreas and frontal lobe, which are known to have low RNA quality post-mortem. (TIF) [file pgen.1004304.s011.tif]

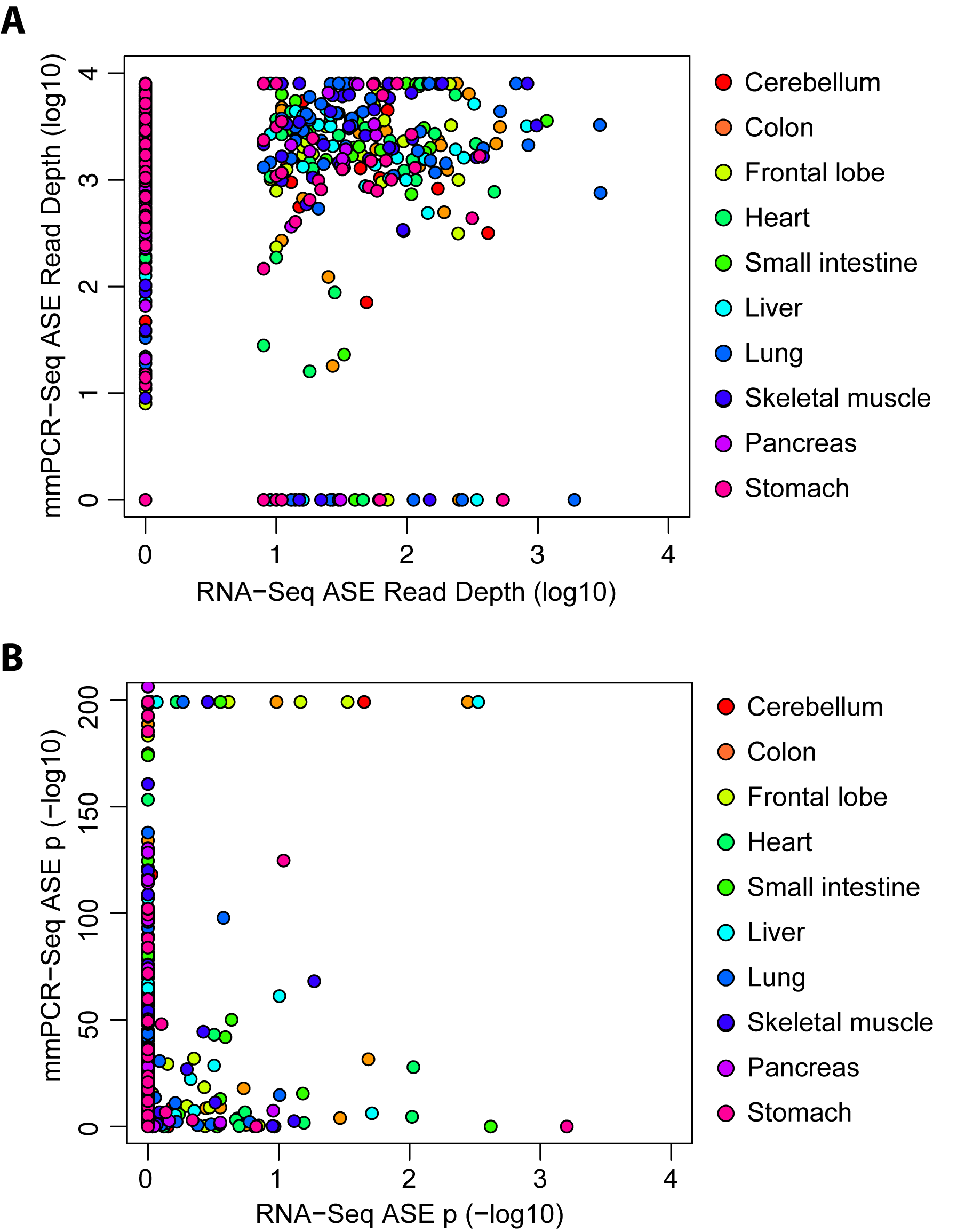

Supplement: Figure S12 — Comparison of coverage of LoF variants using different technologies. (A) Comparison of read depth at heterozygous variants using RNA-Seq and mmPCR-Seq data. The tested heterozygous sites have consistently deeper coverage using mmPCR-Seq. (B) Comparison of ASE detection using RNA-Seq and mmPCR-Seq data. The comparison of p-values obtained from the ASE binomial test for matching heterozygous sites indicates increased enrichment for significant ASE effects using mmPCR-Seq. (TIF) [file pgen.1004304.s012.tif]

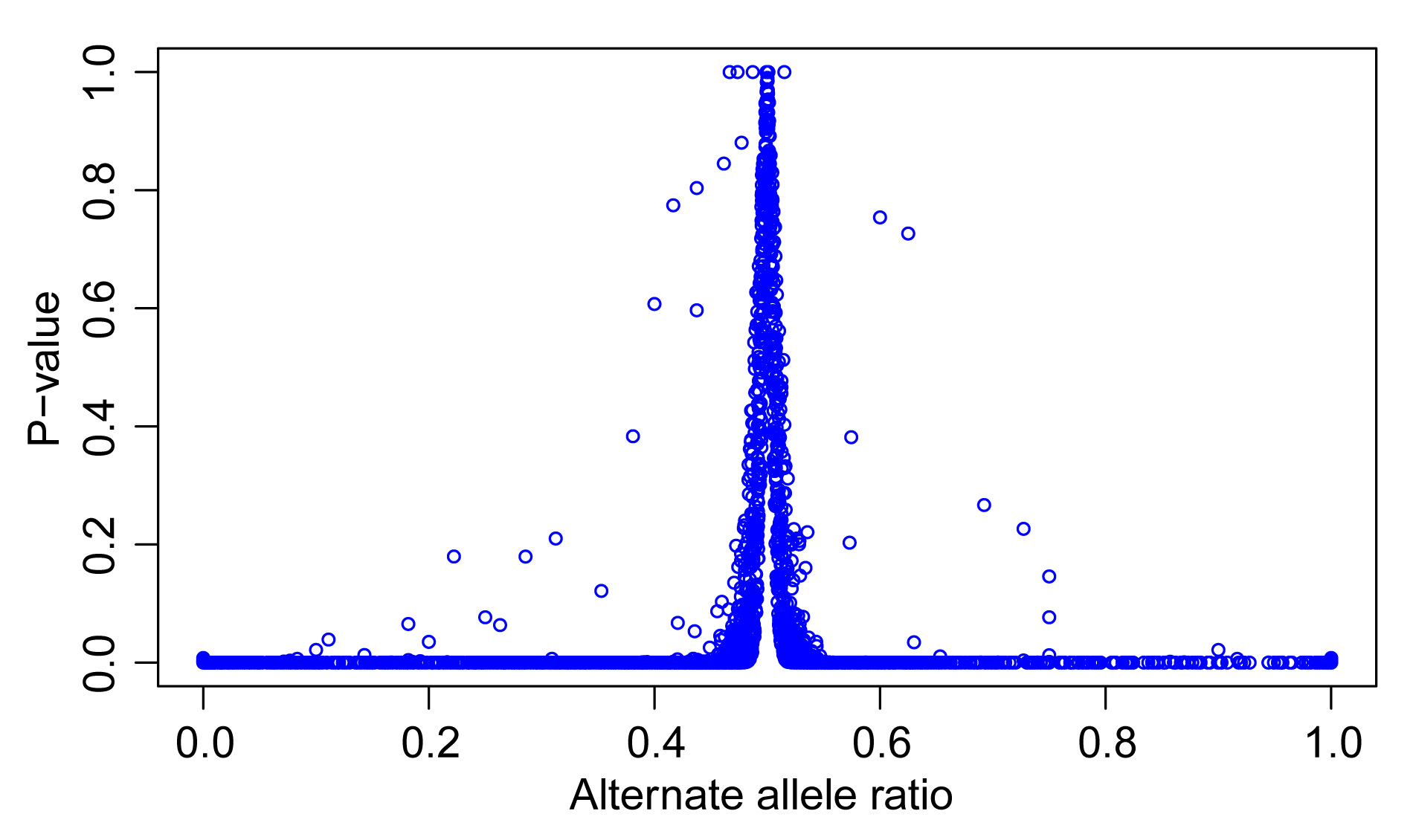

Supplement: Figure S13 — Distribution of alternate allele ratio and corresponding p-value for sites tested by mmPCR-Seq. (TIF) [file pgen.1004304.s013.tif]

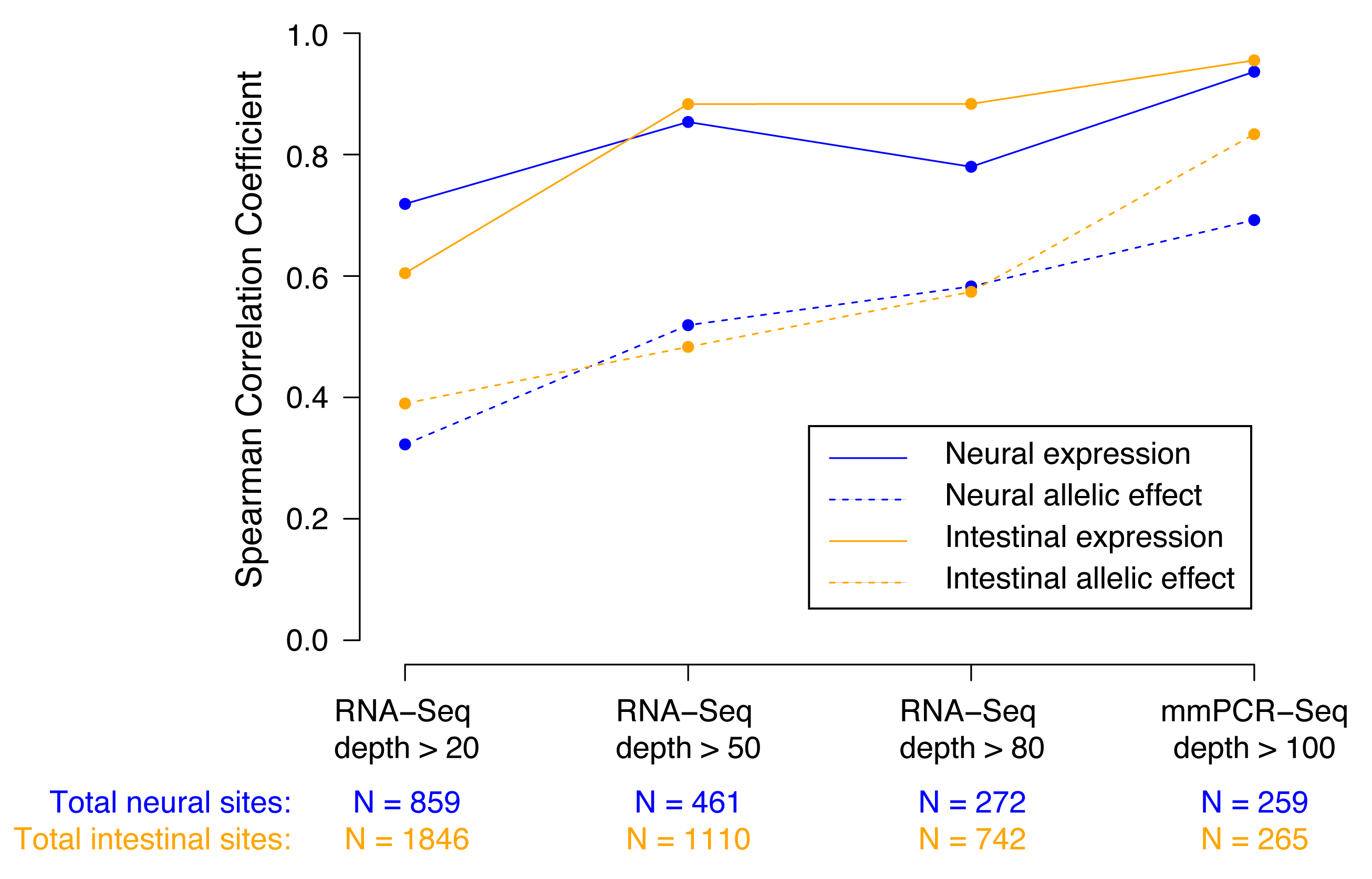

Supplement: Figure S14 — Pairwise correlation of expression and allelic effect for similar tissues. The Spearman correlation coefficient was determined for the pairwise comparisons of neural tissues (frontal lobe and cerebellum) and intestinal tissues (small intestine and colon) for both expression and allelic effect. Independent of read depth, the correlation of expression for tissues of similar embryonic origin are consistently greater than the correlation of allelic effect. (TIF) [file pgen.1004304.s014.tif]

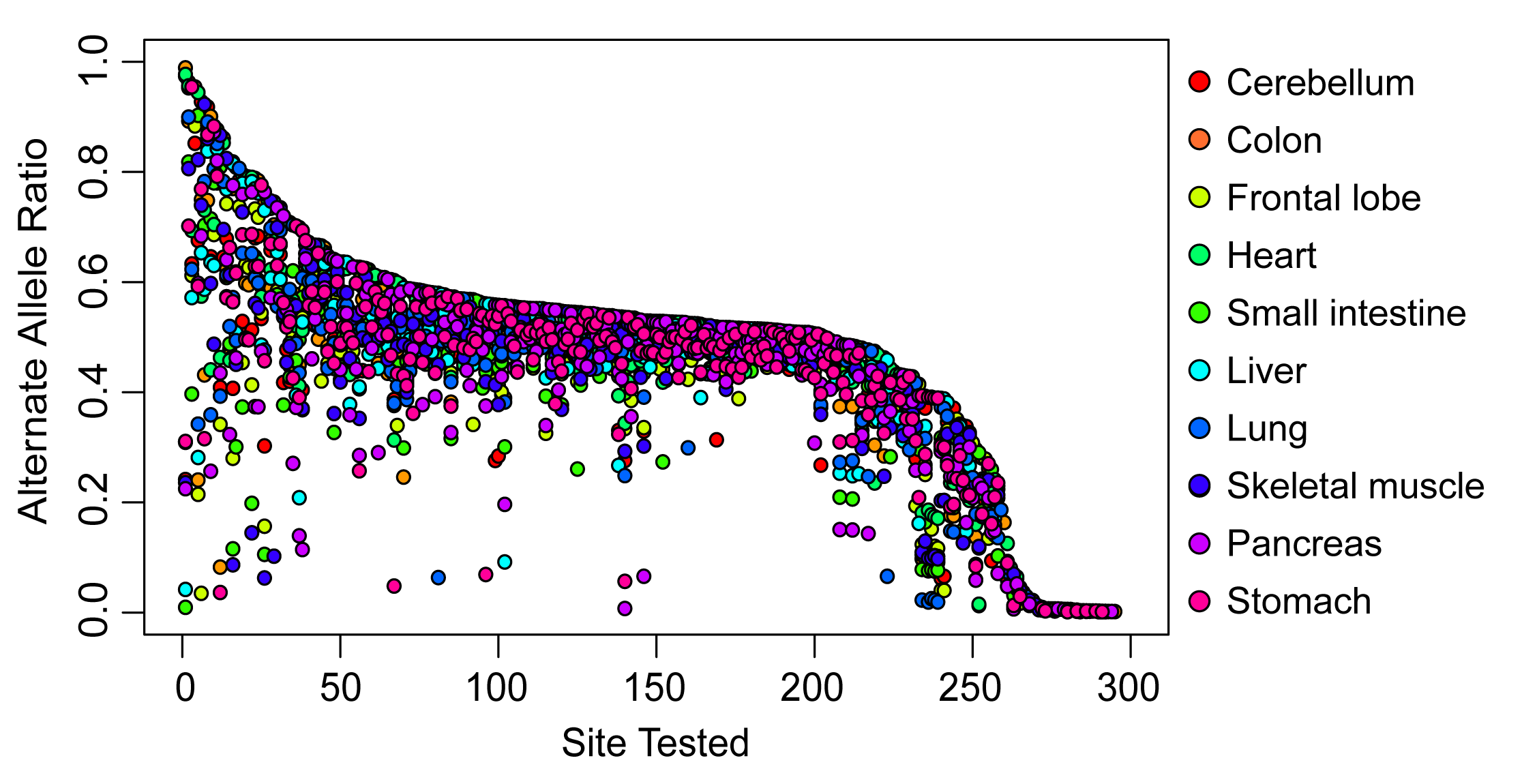

Supplement: Figure S15 — Distribution of alternate allele ratio across tissues from mmPCR-Seq. The alternate allele ratio (alternate allele reads divided by total reads) was calculated for each heterozygous site tested by mmPCR-Seq in all tissues. As expected, the majority of heterozygous sites have an alternate allele ratio of 0.5. For sites with ASE, there appears to be an equal distribution of expression bias towards both the alternate allele (upper left tail) and reference allele (lower right tail). Interestingly, we observed that some sites had measurably varied alternate allele ratios across tissues, while other tested sites had a consistent alternate allele ratio across tissues. (TIF) [file pgen.1004304.s015.tif]

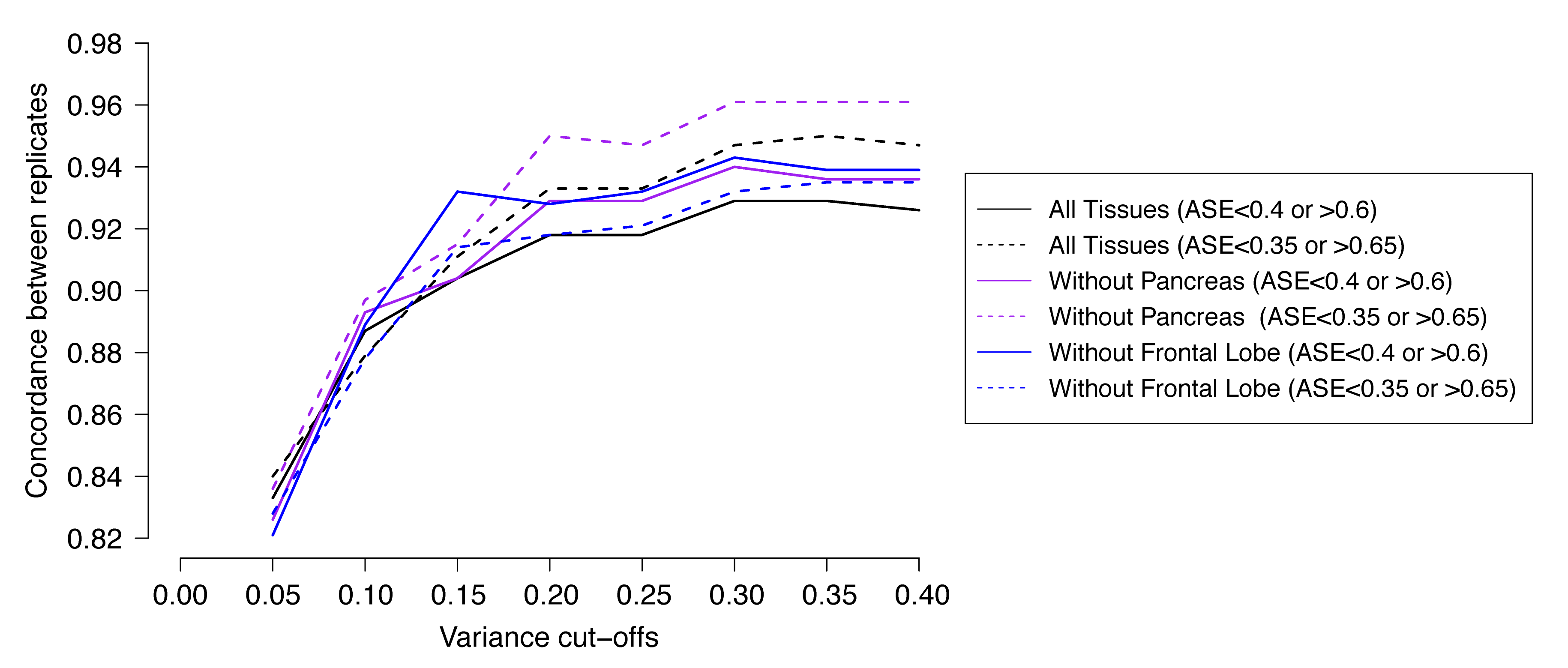

Supplement: Figure S16 — Reproducibility of ASE groups for mmPCR-Seq technical replicates. The reproducibility of the groups (shared ASE, variable ASE, and no ASE) depicted in Figure 3 between replicates was assessed at varying cut-offs. The correlation between replicates was evaluated at two ASE cut-offs (0.4–0.6 and 0.35–0.65) and at eight variance cut-offs (0.05–0.3) for all tissues as well as without the pancreas and frontal lobe. The concordance between replicates increases as the variance cut-off increases and reaches a plateau of ∼95% at a variance of 0.2. The greatest reproducibility is observed when the ASE cutoff is ASE<0.35 or ASE>0.65, the variance cutoff is 0.2, and the pancreas is removed. Using these cut-offs, the reproducibility between replicates for the three groups (non-ASE, shared ASE and variable ASE) is 93.3%. The reproducibility between replicates for the classification of non-ASE and ASE (shared ASE plus variable ASE) is 95.7%. (TIF) [file pgen.1004304.s016.tif]

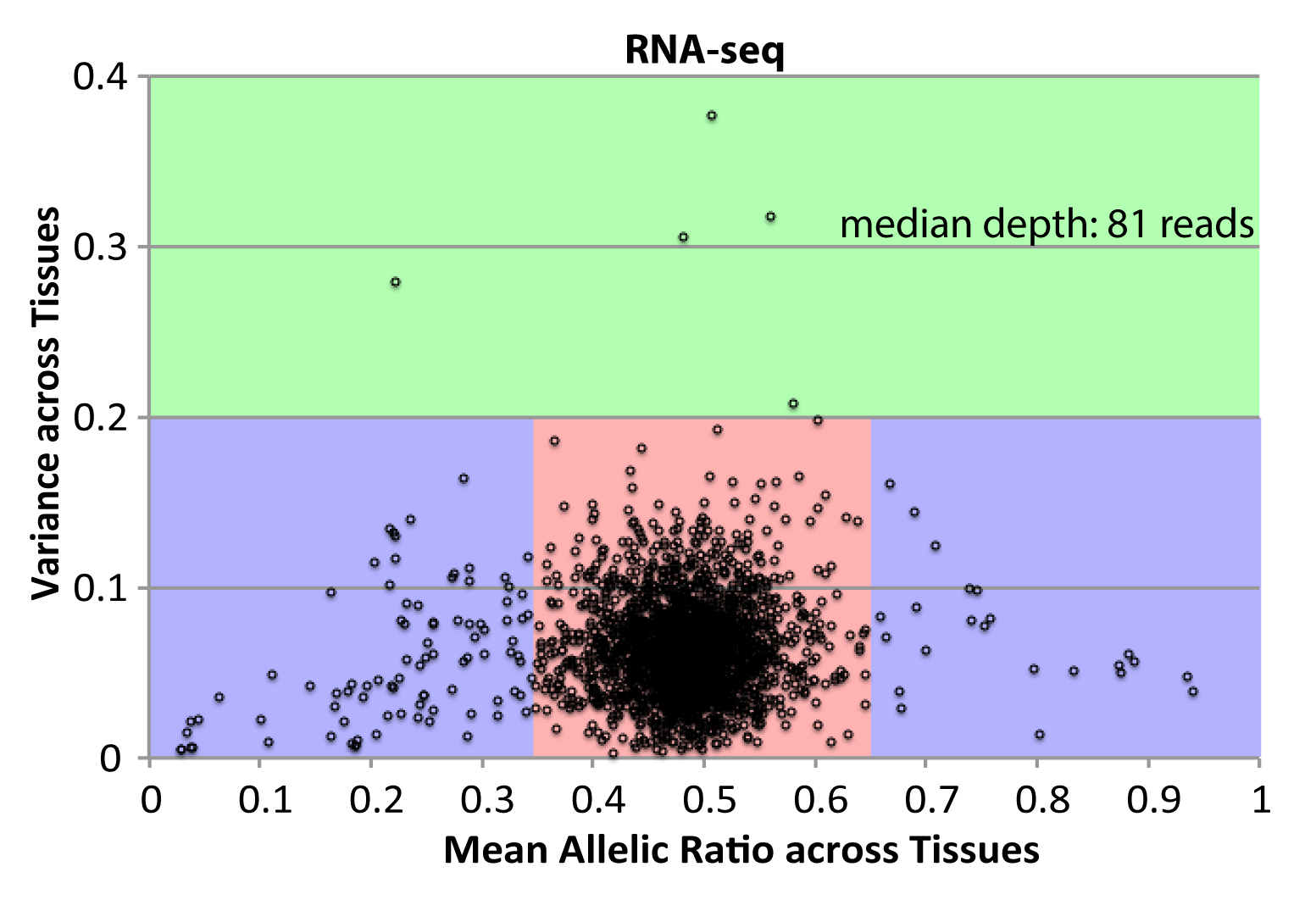

Supplement: Figure S17 — Distribution of shared and variable ASE for RNA-Seq data. The distribution of mean values and standard deviations of the allelic ratios across tissues from the RNA-Seq data. Genomic loci with no ASE and low variance (red), ASE and low variance (blue), and ASE and high variance (green) were divided into three gene groups: no ASE, shared ASE, and variable ASE, respectively. The proportion of sites falling into each ASE group is similar to that found from the mmPCR-Seq data. (TIF) [file pgen.1004304.s017.tif]

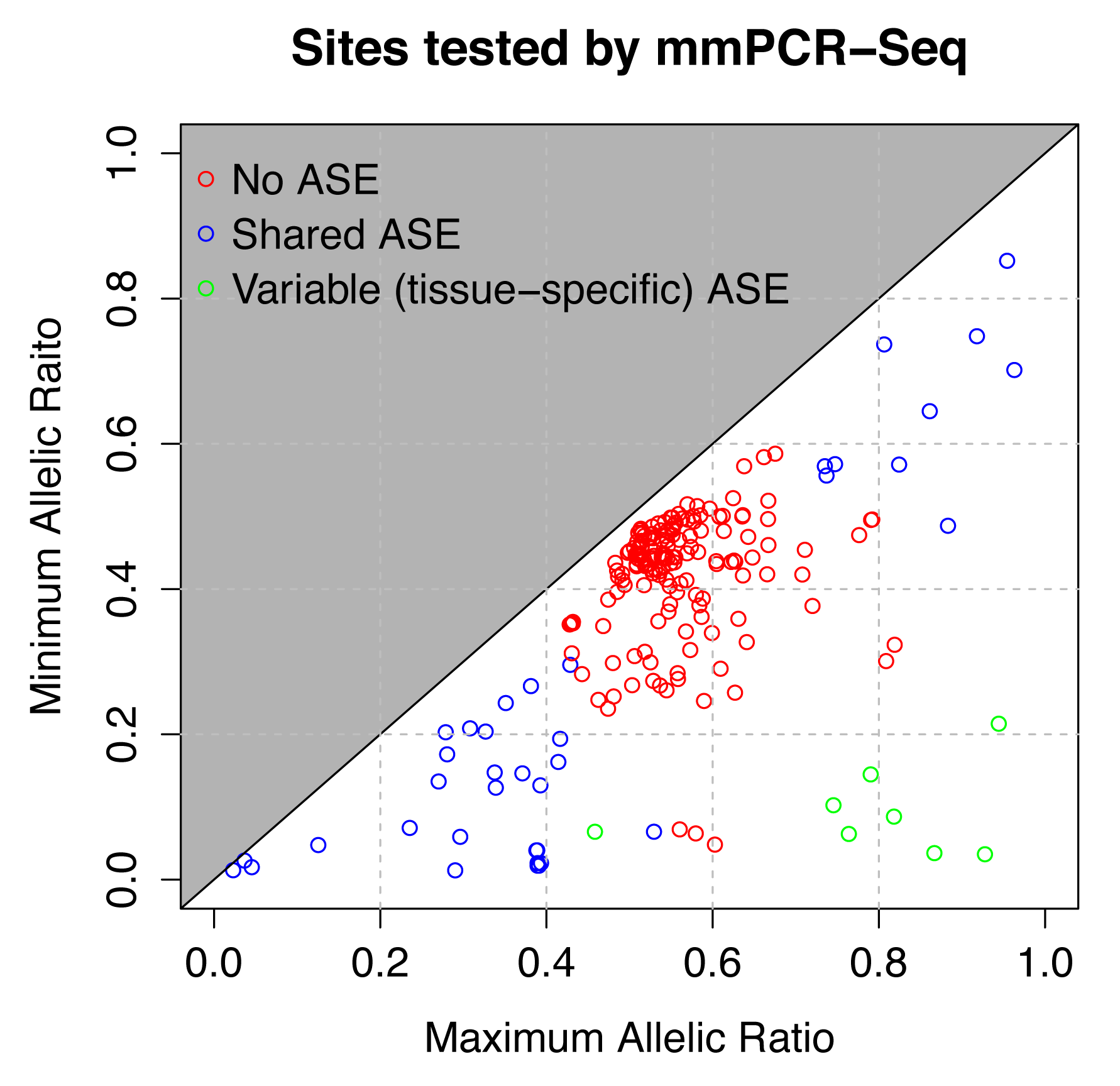

Supplement: Figure S18 — Distribution of effect size and direction of effect for ASE across different tissues. The minimum and maximum alternate allele ratio observed in any tissue for each mmPCR-Seq site tested in at least three tissues are plotted to demonstrate the range of allelic effects observed across tissues. (TIF) [file pgen.1004304.s018.tif]

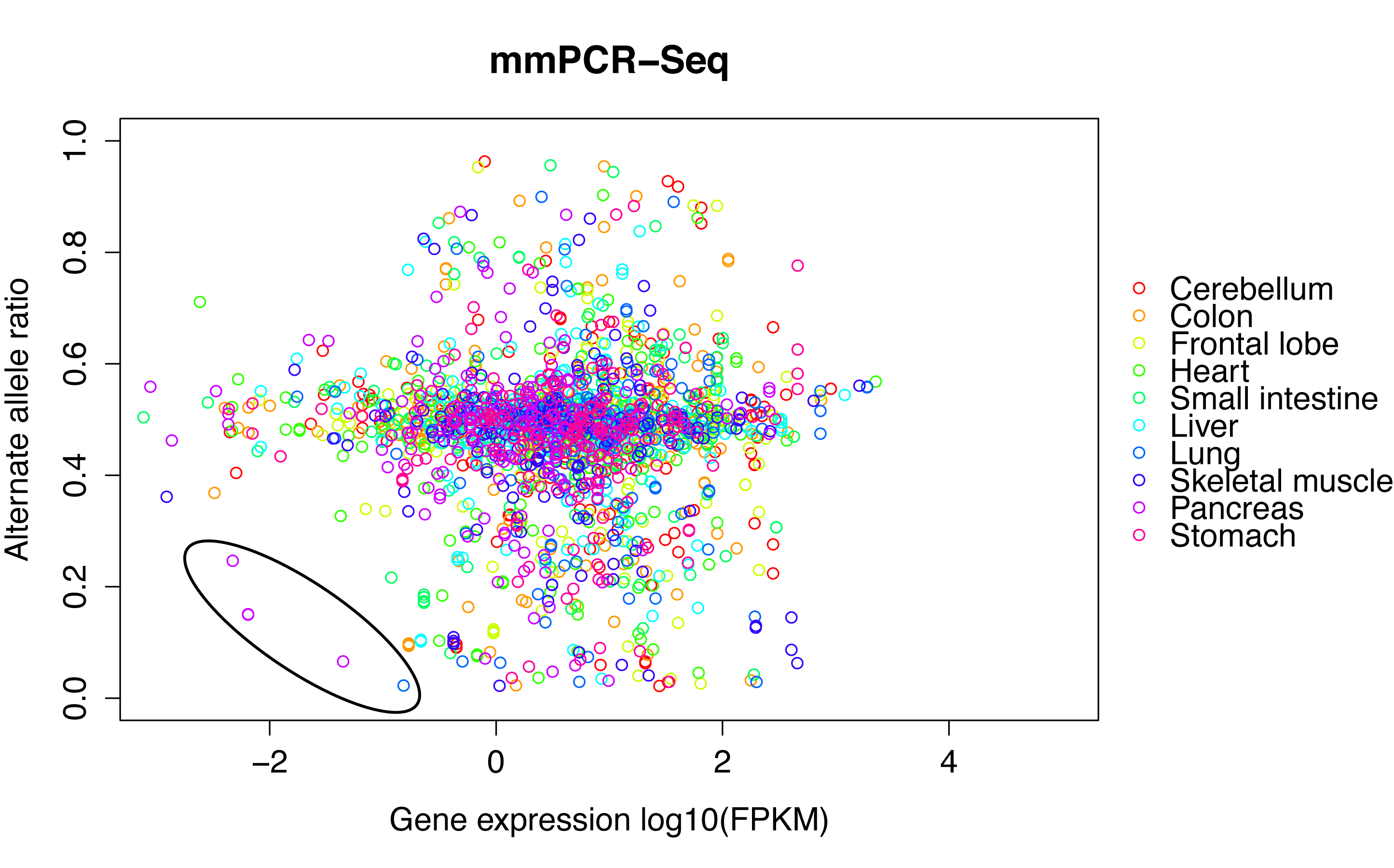

Supplement: Figure S19 — Relationship of ASE effect size and gene expression. The relationship between ASE effect size (measured by mmPCR-Seq) and gene expression level (measured by RNA-Seq) across all tissues was examined. There is no correlation between allelic effect size and gene expression level. Four lowly expressed sites had low allelic ratios (circled) but were not enriched in any class of variants or influenced calling of variable ASE within the study. (TIF) [file pgen.1004304.s019.tif]

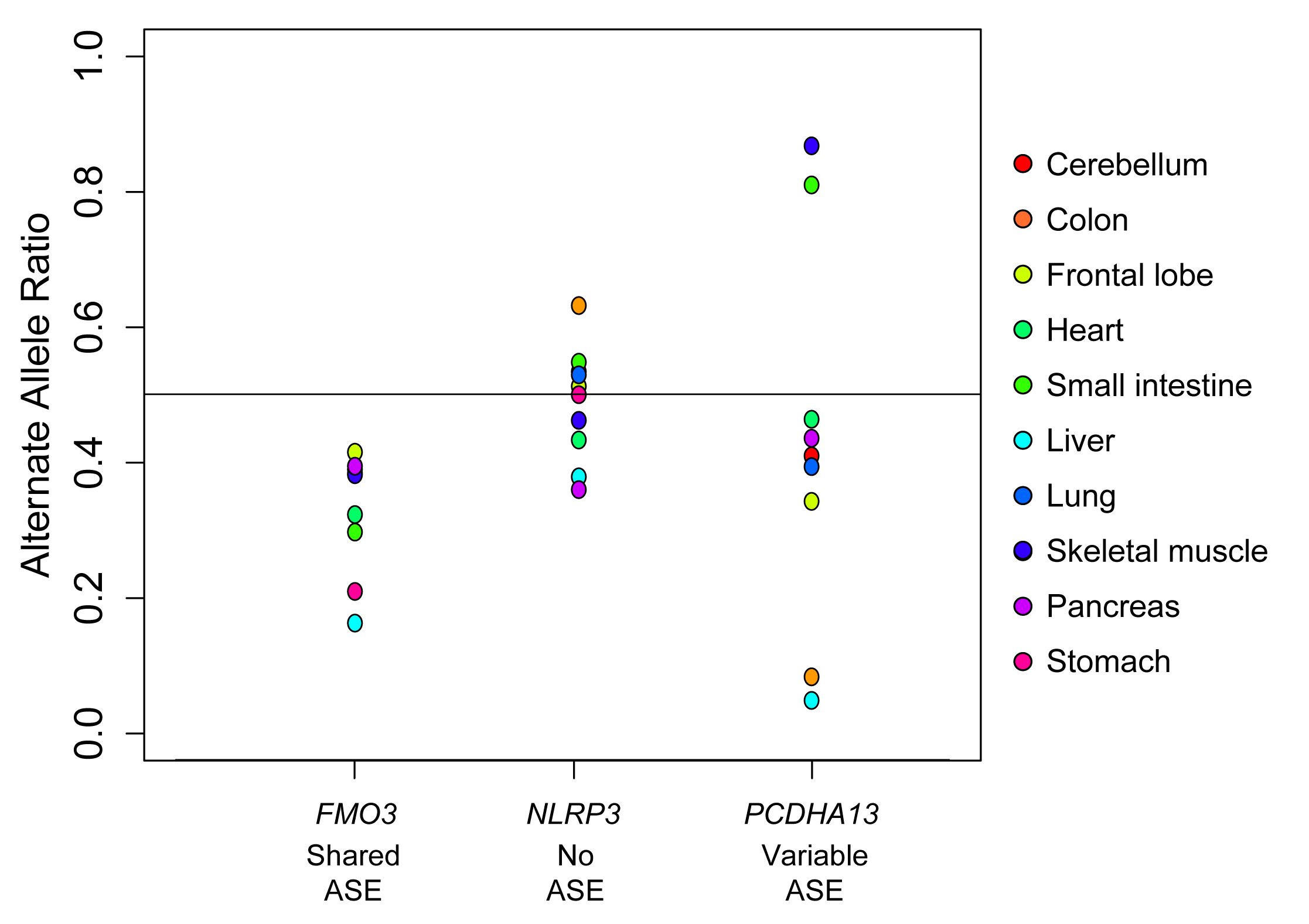

Supplement: Figure S20 — Examples of genes with deleterious nsSNPs exhibiting shared, variable, and no ASE. The gene FMO3, which is associated with the rare Mendelian disorder trimethylaminuria (OMIM 602079), exhibits decreased expression of the deleterious allele across tissues. In contrast, gene NLRP3, which is associated with the Mendelian disease Muckle-Wells Syndrome (OMIM 191900), exhibited no ASE across tissues. The gene PCDHA13, which encodes a protocadherin, is an example of a gene with variable ASE across tissues; the deleterious allele is underexpressed in certain tissues and overexpressed in other tissues. (TIF) [file pgen.1004304.s020.tif]
