# Supplementary material for: Allelic Expression of Deleterious Protein-Coding Variants across Human Tissues
Source: PLoS Genet. 2014 May 1;10(5):e1004304. doi: 10.1371/journal.pgen.1004304 (PMC4006732; doi:10.1371/journal.pgen.1004304)
Supplement: Table S1 — Identification of high-confidence heterozygous and homozygous variants. (PDF) [file pgen.1004304.s021.pdf]

Table S1. Identification of high-confidence heterozygous and homozygous variants

Type	Total Variants	Total High Confidence	Heterozygous High Confidence	Homozygous High Confidence
Total SNVs	51,875	45,058	25,570	19,488
Total gene-associated SNVs	51,829	45,020	25,555	19,465
Total coding	23,100	21,587	12,772	8,815
Missense: benign	9,049	8,476	4,741	3,735
Missense: deleterious (LoF)	2,436	2,124	1,632	492
Nonsense (LoF)	118	105	75	30
Synonymous	11,497	10,882	6,324	4,558
5′UTR	1,403	1,169	593	576
3′UTR	2,130	1,882	1,039	843
Intron	23,539	19,324	10,585	8,739
Ts/Tv	—	2.61	—	—
Rare SNV	5,246	2,767	2,227	540
