# Supplementary material for: Allelic Expression of Deleterious Protein-Coding Variants across Human Tissues
Source: PLoS Genet. 2014 May 1;10(5):e1004304. doi: 10.1371/journal.pgen.1004304 (PMC4006732; doi:10.1371/journal.pgen.1004304)
Supplement: Table S2 — Identification of high-confidence common and rare variants. (PDF) [file pgen.1004304.s022.pdf]

Table S2. Identification of high-confidence common and rare variants

Variant Type	Total High Confidence	Common High Confidence	Rare High Confidence
Total SNVs	45,058	42,291	2,767
Total gene-associated SNVs	45,020	42,253	2,767
Total coding	21,587	20,688	899
Missense: benign	8,476	8,097	379
Missense: deleterious (LOF)	2,124	1,918	206
Nonsense (LOF)	105	96	9
Synonymous	10,882	10,577	305
5′UTR	1,169	1,091	78
3′UTR	1,882	1,788	94
Intron	19,324	17,957	1,367
Ts/Tv	2.61	—	—
