# Supplementary material for: Allelic Expression of Deleterious Protein-Coding Variants across Human Tissues
Source: PLoS Genet. 2014 May 1;10(5):e1004304. doi: 10.1371/journal.pgen.1004304 (PMC4006732; doi:10.1371/journal.pgen.1004304)
Supplement: Table S3 — Identification of high-confident RNA-Seq ASE sites by quality-control filtering. To identify high-confident ASE sites, we implemented several quality control filters. We only kept ASE sites which met the following criteria: 1) base quality (BQ) greater than 10; 2) minimum sequencing depth of 10 reads; 3) calculated p-value less than 0.05 from the binomial test; 4) bi-allelic expression; and 5) intragenic location. (PDF) [file pgen.1004304.s023.pdf]

Table S3. Identification of high-confident RNA-Seq ASE sites by quality-control filtering

Tissue	Ref to Non-ref Ratio	Total sites pre-filters	Total sites post-filters				
			BQ>10	Depth>10	p<0.05	Bi-allelic expression	Intra-genic
Cerebellum	0.553	1,993	1,993	1,802	474	294	277
Colon	0.586	4,045	4,045	3,773	1,148	851	709
Frontal lobe	0.530	3,796	3,796	3,475	779	482	466
Heart	0.554	3,216	3,216	2,954	806	489	458
Small intestine	0.581	3,789	3,789	3,468	1,117	803	683
Liver	0.550	4,215	4,215	3,794	1,177	791	719
Lung	0.587	6,003	6,003	5,550	1,371	945	820
Skeletal muscle	0.561	4,291	4,291	3,900	1,042	649	614
Pancreas	0.594	2,128	2,128	1,839	556	348	332
Stomach	0.623	4,264	4,264	3,826	1,029	680	566

To identify high-confident ASE sites, we implemented several quality control filters. We only kept ASE sites which met the following criteria: 1) base quality (BQ) greater than 10; 2) minimum sequencing depth of 10 reads; 3) calculated p-value less than 0.05 from the binomial test; 4) bi-allelic expression; and 5) intragenic location.
