# Supplementary material for: Allelic Expression of Deleterious Protein-Coding Variants across Human Tissues
Source: PLoS Genet. 2014 May 1;10(5):e1004304. doi: 10.1371/journal.pgen.1004304 (PMC4006732; doi:10.1371/journal.pgen.1004304)
Supplement: Table S4 — OMIM genes associated with Mendelian disease phenotypes harboring rare deleterious nsSNP variants. (PDF) [file pgen.1004304.s024.pdf]

Table S4. OMIM genes associated with Mendelian disease phenotypes harboring rare, deleterious nonsynonymous variants

ASE across tissues	Gene	OMIM disease association
Shared ASE	CACNA1A	Cerebellar ataxia, Hemiplegic migraine
Shared ASE	FMO3	Fish-odor syndrome, Trimethylaminuria,
Shared ASE	TBX21	Asthma and nasal polyps
No ASE	DOCK8	Mental retardation, autosomal dominant
No ASE	MTTP	Abetalipoproteinemia
No ASE	NLRP3	CINCA syndrome, Muckle-Wells syndrome
No ASE	SLC25A13	Citrullinemia type II
No ASE	TMEM168	Alveolar soft-part sarcoma,
