# Supplementary material for: Allelic Expression of Deleterious Protein-Coding Variants across Human Tissues
Source: PLoS Genet. 2014 May 1;10(5):e1004304. doi: 10.1371/journal.pgen.1004304 (PMC4006732; doi:10.1371/journal.pgen.1004304)
Supplement: Table S5 — Allelic imbalance of deleterious nsSNPs, nonsense variants, and control sites across tissues. (PDF) [file pgen.1004304.s025.pdf]

Table S5. Allelic imbalance of deleterious nsSNPs, stop-gained variants, and control sites across tissues

	Control	Deleterious nsSNPs		Stop-gained	
	mean	mean	p-value	mean	p-value
**Cerebellum**	0.481	0.415	0.016	0.391	0.059
**Colon**	0.474	0.430	0.068	0.392	0.057
**Frontal Lobe**	0.465	0.428	0.084	0.363	0.007
**Heart**	0.449	0.432	0.277	0.343	0.016
**Small Intestine**	0.479	0.440	0.065	0.364	0.011
**Liver**	0.460	0.413	0.053	0.343	0.008
**Lung**	0.485	0.412	0.007	0.386	0.025
**Skeletal Muscle**	0.441	0.439	0.463	0.337	0.014
**Pancreas**	0.452	0.394	0.028	0.346	0.031
**Stomach**	0.469	0.438	0.125	0.359	0.015

We tested if deleterious nsSNPs and stop-gained variants have a statistically significant reduction in expression across all tissues compared to control sites. We calculated the mean allelic ratio for each variant group and used the student’s t-test to determine if there is a significant difference between the control sites and the corresponding variant group (deleterious nsSNPs or stop-gained variants). The mean allelic ratios for each variant group and the calculated p-values are shown in the table.
